# Epigenetic Characteristics of Human Subtelomeres Vary in Cells Utilizing the Alternative Lengthening of Telomeres (ALT) Pathway

**DOI:** 10.3390/life11040278

**Published:** 2021-03-26

**Authors:** Shir Toubiana, Aya Tzur-Gilat, Sara Selig

**Affiliations:** 1 Department of Genetics and Developmental Biology, Rappaport Faculty of Medicine and Research Institute, Technion, Haifa 31096, Israel; shir.toubiana@gmail.com (S.T.); ayagilat@gmail.com (A.T.-G.); 2 Laboratory of Molecular Medicine, Rambam Health Care Campus, Haifa 31096, Israel

**Keywords:** alternative lengthening of telomeres (ALT), telomere, subtelomere, DNA methylation, TERRA, H3K4me3, H3K9me3

## Abstract

Most human cancers circumvent senescence by activating a telomere length maintenance mechanism, most commonly involving telomerase activation. A minority of cancers utilize the recombination-based alternative lengthening of telomeres (ALT) pathway. The exact requirements for unleashing normally repressed recombination at telomeres are yet unclear. Epigenetic modifications at telomeric regions were suggested to be pivotal for activating ALT; however, conflicting data exist regarding their exact nature and necessity. To uncover common ALT-positive epigenetic characteristics, we performed a comprehensive analysis of subtelomeric DNA methylation, histone modifications, and TERRA expression in several ALT-positive and ALT-negative cell lines. We found that subtelomeric DNA methylation does not differentiate between the ALT-positive and ALT-negative groups, and most of the analyzed subtelomeres within each group do not share common DNA methylation patterns. Additionally, similar TERRA levels were measured in the ALT-positive and ALT-negative groups, and TERRA levels varied significantly among the members of the ALT-positive group. Subtelomeric H3K4 and H3K9 trimethylation also differed significantly between samples in the ALT-positive group. Our findings do not support a common route by which epigenetic modifications activate telomeric recombination in ALT-positive cells, and thus, different therapeutic approaches will be necessary to overcome ALT-dependent cellular immortalization.

## 1. Introduction

Telomeres are structures that cap all eukaryotic chromosome ends. Human telomeric DNA at birth consists of approximately 12 kb of TTAGGG repeats [1], complexed mainly with the protective Shelterin proteins [2]. Following terminal differentiation, human telomeres shorten continuously with each cell division, and when they reach a critically short length, cells enter senescence [3]. Telomere length-associated senescence, termed also as replicative senescence, fulfills a tumor-suppressing function, since shortening beyond a critically short length in cells with properly functioning cellular checkpoints, results in chromosome ends recognized as double-strand DNA breaks (DSB) [4]. Continuous cell divisions in the presence of critically short telomeres, as occurs frequently during malignant transformation, induce chromosome end fusions that lead to major genomic instability, one of the hallmarks of cancer [5]. To avoid replicative senescence, the vast majority of human cancers activate a mechanism that ensures telomere length maintenance. Approximately 75–90% of human cancers activate the expression of the human telomerase reverse transcriptase (*hTERT*) gene [6,7], while most, but not all [8], of the remaining human cancers maintain telomere length by the alternative lengthening of telomeres (ALT) pathway. The hallmarks of the ALT pathway include heterogenous telomere length, the presence of modified telomeric repeats, abnormally high levels of telomere–sister chromatid exchange (T-SCE), extrachromosomal telomeric repeats (ECTR), and ALT-associated PML bodies (APBs) (reviewed in [8,9,10]). The majority of the human ALT cell lines maintain their telomeres by a mechanism reminiscent of that in RAD52-dependent yeast type II survivors (reviewed in [10]). Similar to yeast, this mechanism depends on break-induced replication (BIR), which is mediated by conservative replication and requires the activity of the PolD3 and PolD4 subunits of DNA polymerase delta [10,11]. The striking heterogeneity in telomere lengths in ALT cells arises from homologous recombination (HR) between telomeric repeats, mostly between telomeres of independent chromosome ends. HR at telomeres is normally suppressed, and the exact mechanism/s by which this repression is overruled in ALT cancers is yet unclear [10,12]. 

One of the mechanisms proposed to unleash HR at ALT telomeres involves epigenetic changes at telomeres and subtelomeres (reviewed in [13]). Subtelomeres are the regions immediately adjacent to the telomeric repeats. The most distal human subtelomeric regions are extremely CpG-rich, normally highly methylated, and many subtelomeres contain promoters for the long non-coding telomeric repeat-containing RNA (TERRA) [14,15]. Many physiological roles have been ascribed to TERRA, among them replication of telomeric regions and regulation of telomeric chromatin and telomerase activity [16,17]. In ALT cancers, it was reported that TERRA levels are elevated due to DNA hypomethylation of subtelomeric regions [18,19]. The elevated TERRA levels were demonstrated to generate telomeric DNA:RNA hybrids, which in turn facilitate HR at telomeric regions [19]. However, a series of studies that investigated subtelomeric DNA methylation in ALT-positive (ALT+) versus telomerase-positive ALT-negative (ALT-) cells reported conflicting findings regarding the extent of subtelomeric hypomethylation, which depended on the specific ALT+ cell analyzed [18,19,20,21,22]. It is worth noting that the studied ALT+ cells were not always of tumor origin [22], and the regions studied for DNA methylation did not always overlap with TERRA promoter regions [20,21]. Moreover, there is no consensus regarding the requirement of subtelomeric hypomethylation for HR at telomeres in ALT+ cells [18,20,21]. While additional studies support the role of telomeric DNA:RNA hybrids in generating the ALT phenotype [23,24,25], it is unclear how many telomeres contribute to the high TERRA levels, and whether TERRA in ALT+ cells acts both in *cis* and in *trans* to generate DNA:RNA hybrids.

Understanding the molecular basis of telomere maintenance in ALT+ cancers is crucial for devising therapeutic tools for this class of cancers, as well as for telomerase-positive cancers that escape anti telomerase-based therapies by activating ALT [12,26]. Several strategies have been proposed for targeting ALT+ tumors. One promising approach is ATR inhibition that should interfere with the replication stress response, which is central for activating ALT. Disruption of APBs, and thus abolishing ALT activity, is another strategy predicted to prevent the lengthening of the shortest telomeres in the cell and lead to senescence or cell death. Additionally suggested approaches include targeting of ALT specific telomere proteins or inhibiting the specialized form of HR that occurs in ALT+ cells (reviewed in [10,12]). Altered chromatin traits in ALT+ cells are implicated in setting the stage for the HR typical for this tumor type. Therefore, we performed a comprehensive analysis of several epigenetic characteristics of subtelomeric regions in ALT+ and ALT- cell lines to determine to what extent ALT+ cells share specific epigenetic features. Our findings indicate that subtelomeric DNA methylation, TERRA expression levels, and specific subtelomeric histone modifications are highly variable between ALT+ cell lines.

## 2. Materials and Methods

### 2.1. Cell Lines and Tissue Culture

ALT+ and ALT- cell lines utilized in this study appear in Table 1. A-431, MCF7, SUSM-1, and VA-13 were cultured in DMEM supplemented with 10 % FCS, glutamine, and antibiotics. G-292, MDA-MB-468, OVCAR-3, Saos-2, SK-OV-3, and U-2 OS were cultured in RPMI-1640 supplemented with 10 % FCS, glutamine, and antibiotics. SK-LU-1 and H295R were cultured according to the conditions specified by ATCC. Control cell lines included the Immunodeficiency, Centromeric instability and Facial anomalies type 1 (ICF1) lymphoblastoid cell line (LCL), GM8714 (Coriell Institute) cultured in RPMI-1640 supplemented with 15 % FCS, glutamine and antibiotics, fibroblast-like cells (FLs) derived from induced pluripotent stem cells (iPSCs) of ICF1 patient pR [27], and wild-type primary fibroblasts FSE and UN [28]. All fibroblasts were cultured in DMEM supplemented with 10 % FCS, glutamine, and antibiotics.

### 2.2. Terminal Restriction Fragment (TRF) Analysis

Genomic DNA was extracted according to standard procedures and treated with RNase A. *Hinf-I* digested DNA was separated on a 0.7% agarose gel and transferred to a charged nylon transfer membrane (1226556, GVS North America, Sanford, ME, USA) using a BIO-RAD vacuum blotter. Membranes were hybridized to a telomeric probe and washed as described [31].

### 2.3. DNA Methylation Analysis by Southern Blots

To study subtelomeric methylation, TRF analysis was performed as described above following the digestion of DNA samples with each of the two isoschizomeric restriction enzymes: *HpaII* (methylation-sensitive enzyme) and *MspI* (methylation-insensitive enzyme). Methylation analyses of satellite 2 and NBL-1 repeats were performed as previously described [32,33].

### 2.4. DNA Methylation Analysis by Targeted Bisulfite Sequencing

Genomic DNA (0.5–1 µg) was bisulfite-converted with the Methylamp DNA Modification kit (P-1001, EPIGENTEK, Farmingdale, NY, USA). Following conversion, DNA was amplified using FastStart Taq DNA Polymerase, dNTPack (04738314001, Sigma-Aldrich, St. Louis, MO, USA) using the followed amplification program: 95 °C for 5 min; 4 cycles of 95 °C for 1 min, 53 °C for 3 min, 72 °C for 3 min; 2 cycles of 95 °C for 30 s, 55 °C for 45 s, 72 °C for 45 s; 40 cycles of 95 °C for 30 s, 72 °C for 1.5 min; 72 °C for 10 min. Primers for amplification of satellite 2 and subtelomeric regions were described previously [27,28]. PCR products were purified by the QIAquick PCR purification kit (28104, Qiagen, Hilden, Germany), quantified, diluted to 10 pM, pooled into one tube, and subjected to emulsion PCR (E-PCR) on ion sphere particles (ISPs; Thermo Fisher Scientific, Waltham, MA, USA) using the Ion PGM TM Sequencing 400 Kit on the Ion OneTouch system (Thermo Fisher Scientific). Reaction efficiency was estimated by calculating the percentage of DNA-containing ISPs compared to the background of blank ISPs using the Qubit Ion Sphere quality control kit (Thermo Fisher Scientific). Enriched ISPs were subjected to sequencing on the Ion 314 Chip using the Ion PGM^TM^ Hi-Q^TM^ Sequencing Kit. The multiple reads (1200–28,700) generated for each amplicon were aligned to the target sequences, after which FASTQ files were generated. Following alignment, the percent of methylation for each CpG site was calculated for 100 to 6100 analyzed reads using Bismark software (Babraham Institute website) [34].

### 2.5. RT-qPCR Analysis 

RT-qPCR TERRA expression analysis was performed only for subtelomeres with clear telomeric ends [15]. RNA was isolated using the RNeasy Mini Kit (74104, Qiagen). The purified RNA was treated with TURBO DNase (AM2238, Thermo Fisher Scientific) to eliminate any trace of DNA. For expression of coding genes, cDNA was prepared from 2 μg RNA using the 5X All-in-One MasterMix (G485, Applied Biological Material, Inc., Richmond, BC, Canada). For TERRA expression, 1 µg RNA was reverse transcribed at 55 °C using SuperScript III reverse transcriptase (Invitrogen), a β-actin-specific primer (5′-AGTCCGCCTAGAAGCATTTG-3′), and a TERRA-specific primer composed from five telomere-hexameric repeats (CCCTAA)_5_, as described [35]. RT-qPCR was performed with Fast SYBR Green Master Mix (4385612, Thermo Fisher Scientific), on an Applied Biosystems StepOnePlus Real-Time PCR system (AB-4385612, Thermo Fisher Scientific). Primers for amplification of cDNA appear in Tables S1 and S2. The primers for amplification of TERRA from different chromosome ends were designed to anneal to regions distal to putative TERRA transcription start sites [36]. RT-qPCR values were normalized to the human *HPRT1* transcript for the protein-coding genes and the human *β-actin* transcript for subtelomeric-specific TERRA, by the 2^−ΔΔCT^ method. Authentic expression of TERRA was based on melting curves, which exhibited a clear single peak as described [37,38]. In addition, we amplified TERRA from ICF1 control cells, which produce a clear melting curve due to high levels of TERRA expression [39], and verified that the melting peaks obtained from amplification of ALT+, ALT-, and WT cDNA samples form at an identical position as that in the ICF1 sample.

### 2.6. Chromatin Immunoprecipitation (ChIP) Analysis 

ChIP analyses were performed as described previously [27]. Antibodies used for immunoprecipitation included anti-H3K4me3 (ab8580, Abcam, Cambridge, UK) and anti-H3K9me3 (ab8898, Abcam). Normalization was done relative to an amplified negative region in the same sample (Hoxa7 TSS and GAPDH promoter for H3K4me3 and H3K9me3, respectively) to exclude technical variations between samples. At first, normalization to the input amount was calculated, and then, the obtained percentage of input was used to calculate the enrichment over the percentage of input of the negative control region. All primers used for ChIP analyses appear in Table S2.

### 2.7. Statistical Analyses

All statistical analyses were performed using Python 2.7 and the Python libraries “numpy”, “scipy”, “statsmodels”, “pandas”, “jupyter”, and “notebook”. “Matplotlib” and “seaborn” were used for graphics. For the methylation analysis, the Pearson correlation test was used to determine the correlation between sample pairs to classify the samples into clusters. The two data vectors that served as test inputs were composed of methylation percentages at all corresponding CpG positions in all subtelomeres of the two compared samples. In the case of failure to amplify a particular amplicon, it was excluded from the vector of both compared samples. For gene expression, TERRA expression, and ChIP analyses, bars and error bars represent means and ± standard error of means (SEM), respectively, based on at least three independent experiments. Statistical analyses of TERRA expression were done using Welch’s unequal variances *t*-test (two-tails). For gene expression and ChIP, statistical analyses were done using the non-parametric Mann–Whitney U-test (two tails).

## 3. Results

### 3.1. Cell Lines Selected for Study

To examine whether certain epigenetic properties are common for all cell lines that activate the ALT pathway for telomere maintenance, we studied seven ALT+ cell lines, of which five were established from tumors, and two were generated from normal fibroblasts by chemical or SV40 transformation [40] (Table 1). TRF analysis validated the typical hybridization pattern for ALT-maintained telomeres (Figure S1a) and [41,42]. Of the five ALT- cell lines studied here (Table 1), all but SKOV-3 demonstrated very short telomere length, and none showed the large variation in telomere length apparent in the ALT+ cell lines (Figure S1b).

### 3.2. DNMT3B Is Transcribed at Lower Levels in ALT+ Cell Lines

**Figure 1:**
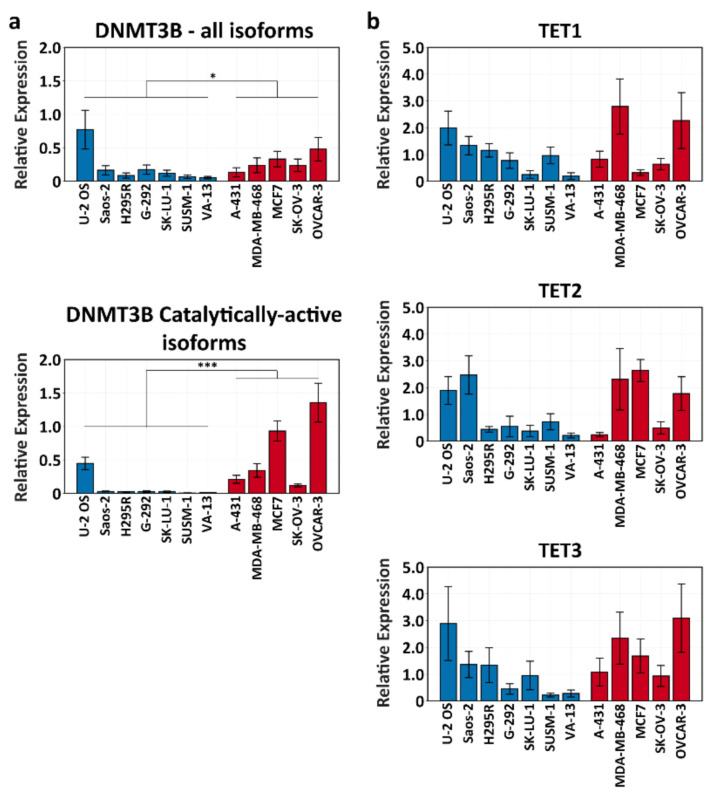
Expression of *DNMT3B* and *TET* family members in ALT+ and ALT- cell lines. (**a**) RT-qPCR analysis of *DNMT3B* in ALT+ (blue) and ALT- (red) cell lines. The analysis was performed with two primer pairs: one that amplifies a region common to all isoforms (top), and a second that amplifies a region specific for the catalytically active isoforms (bottom). Bars and error bars represent means and ± standard error of means (SEM), respectively, based on at least two independent experiments. (**b**) RT-qPCR analysis of *TET1, TET2*, and *TET3* genes in ALT+ (blue) and ALT- (red) cell lines. Bars and error bars represent means and ± standard error of means (SEM), respectively, based on at least two independent experiments. For both (**a**) and (**b**), significant differences in the expression level of each amplicon within the members of each group (Tables S3 and S4, respectively) and between both groups collectively was tested for by a two-tailed Mann–Whitney U-test (*p* < 0.05*, *p* < 0.001***).

Subtelomeric hypomethylation in ALT+ cell lines was demonstrated previously [19,20], however no significant differences in the expression of DNA methyltransferases (DNMT) were detected between ALT+ and ALT- cell lines [21]. DNMT3B is a DNMT that specifically targets repetitive regions, among them subtelomeres, and its loss of function leads to subtelomeric hypomethylation in ICF1 syndrome [31,43]. *DNMT3B* generates multiple isoforms, of which only two, *DNMT3B1* and *DNMT3B2*, include the catalytic domain of the enzyme [44]. However, the specific expression of these catalytically active isoforms was not examined previously in ALT+ cells [21]. We, therefore, reevaluated the expression of *DNMT3B* in ALT+ vs. ALT- cells by amplifying both a region common to all isoforms and a region specific only for the catalytically active isoforms (Figure 1a). Our analysis revealed a significant difference in the expression level of the common isoform between both groups (*p* < 0.05). However, the difference in the expression level of the catalytic isoforms was even greater (*p* < 0.001). In both cases, the expression was lower in the ALT+ group. This finding suggested that a relative decrease in the levels of catalytically active DNMT3B could lead to hypomethylation of its target regions, among them subtelomeric regions. Nevertheless, it should be noted that when we compared the samples within each of the groups, significant differences were apparent in the expression level of the catalytic isoforms in the ALT+ group (Table S3).

DNA methylation is also regulated by active demethylation carried out by enzymes from the ten-eleven translocation methylcytosine dioxygenases (TET) family of proteins, and mutations in *TET* genes have been implicated in hypomethylated states in various cancers [45,46]. To determine whether the expression of TET family members is dysregulated in ALT+ cell lines, we determined the expression of the three *TET* genes in the ALT+ and ALT- cell lines (Figure 1b). However, a comparison between both groups did not reveal a significant difference in the expression of the three different *TET* genes. In addition, the members within each of the two groups did not differ significantly for any of the *TET* family transcripts (Table S4).

### 3.3. Subtelomeric DNA Methylation Patterns and Levels Do Not Differentiate between ALT+ and ALT- Cell Lines

Following our findings of decreased levels of catalytically active *DNMT3B* isoforms in the ALT+ group, we next asked whether these findings are associated with significant differences in subtelomeric methylation between both groups. A modified TRF analysis utilizing the isoschizomeric restriction enzymes *HpaII* and *MspI*, sensitive and insensitive to DNA methylation, respectively, allows the study of the methylation status for all subtelomeric regions collectively [31]. Using this method of analysis on four ALT- cell lines (Figure S2a), we found that OVCAR-3 and A-431 cell lines showed a higher molecular weight smear following *HpaII* compared to *MspI* restriction, hence more subtelomeric methylation, in comparison to SK-OV-3 and MCF7, which were less methylated. None of these cell lines showed drastic subtelomeric hypomethylation, as found in the ICF1 syndrome hypomethylated control (Figure S2a). However, this type of analysis is unsuitable for ALT-positive cell lines because of the large variation in telomere length and the subset of very long telomeres (Figure S2b). To circumvent this problem, we performed Southern analysis with the Hutel subtelomeric probe, which anneals to a TERRA promoter region [28]. Similar to the modified TRF analysis, subtelomeric methylation can be deduced by comparing the hybridization patterns that appear following digestion with the isoschizomeric enzymes *Sau3AI* and *MboI* (sensitive and insensitive to methylation, respectively). This analysis indicated that ALT- cell lines are not equally methylated at the subtelomeric regions detected by the Hutel probe (Figure S2c). Some lines, such as MCF7, showed high DNA methylation of this region similar to that found in cord blood DNA (Figure S2e), while some such as OVCAR-3 were partially hypomethylated. In contrast, all five examined ALT+ cell lines demonstrated various degrees of hypomethylation of this subtelomeric region (Figure S2d).

**Figure 2:**
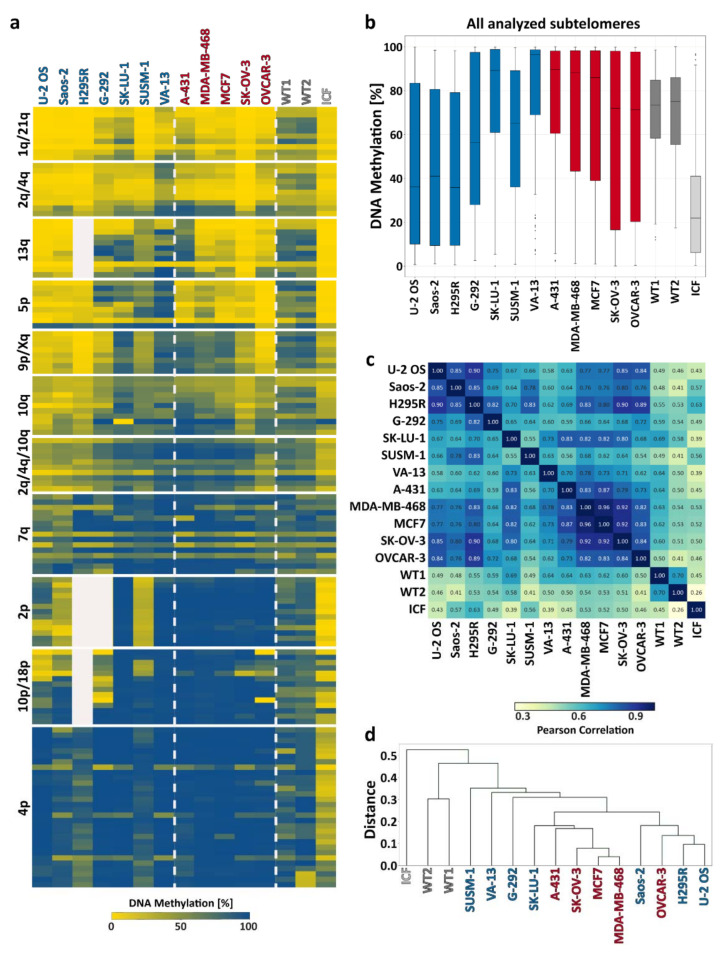
Subtelomeric methylation patterns in ALT+ and ALT- cell lines. Subtelomeric methylation was analyzed by high-throughput sequencing of PCR-amplified bisulfite-converted DNA at 14 subtelomeric regions in ALT+ and ALT- cell lines compared to WT fibroblasts (WT1—UN, WT2—FSE) and ICF1 fibroblast-like cells. (**a**) A heat map illustrating the percentage of methylation across various amplicons. Each amplicon consists of several lines, each line representing a specific CpG site, in the 5′ to 3′ direction (top to bottom). (**b**) Box plots presenting the methylation percentage distribution of all CpG sites of all amplicons for each sample (ALT+ appear in blue, ALT- in red, WT fibroblasts and ICF fibroblast-like cells in gray shades). (**c**) A Pearson correlation analysis based on data described in (**a**) was performed using methylation percentages at each of the CpG sites in the corresponding amplicons of each two compared samples. For regions that failed to amplify (2p for H295R and G-292, 13q and 10p/18p for H295R), the test excluded these amplicons in both compared samples. The degree of similarity is depicted by the cell color and by the values in the cells. Values represent the linear correlation coefficient between the two compared samples. Values close to 1 signify high similarity between the compared samples. (**d**) Hierarchical cluster analysis based on the Pearson correlation analysis described in (**c**).

To evaluate subtelomeric methylation in a quantitative manner, we performed targeted bisulfite analysis of several subtelomeric regions. Most of the analyzed regions are part of canonical TERRA promoters that become hypomethylated when DNMT3B function is impaired [27]. A total of 14 subtelomeres were inspected in both ALT+ and ALT- cell lines and in control cells consisting of two WT and one ICF1 fibroblast cell lines (Figure 2a,b). Pearson correlation analysis of the bisulfite analysis indicated that in the ALT+ group only U-2 OS, Saos-2, and H295R are similar to each other and are distinct from WT fibroblast samples. However, the remaining ALT+ samples do not display high similarity (Figure 2c). The same is true for the ALT- group, which demonstrates similarity only between MDA-MB-468, MCF7, and SK-OV-3 samples (Figure 2c). Hierarchical cluster analysis of all the samples, based on the Pearson analysis, demonstrates the above-mentioned clustering of only a subset of samples within each of the different groups and highlights the lack of uniformity in subtelomeric methylation within both ALT+ and ALT- groups (Figure 2d). This analysis also demonstrates that OVCAR-3, an ALT- cell line, clusters with three ALT+ cell lines rather than with the remaining ALT- samples, and SK-LU-1, an ALT+ cell line, clusters with several ALT- cell lines rather than with ALT+ cell lines. Moreover, it is noticeable by observing the heatmap (Figure 2a) that methylation levels of several subtelomeres, such as 9p/Xq and 10q, vary considerably within each of the groups or only within the ALT+ group, such as in the case of 2p and 10p/18p. Interestingly, both the ALT+ and ALT- groups demonstrated higher variation in methylation percentages among all analyzed subtelomeric regions compared to the WT fibroblasts, which were 60–80% methylated at all amplicons (Figure 2b). In summary, we could conclude that subtelomeric methylation does not distinguish between the ALT+ and ALT- groups. 

**Figure 3:**
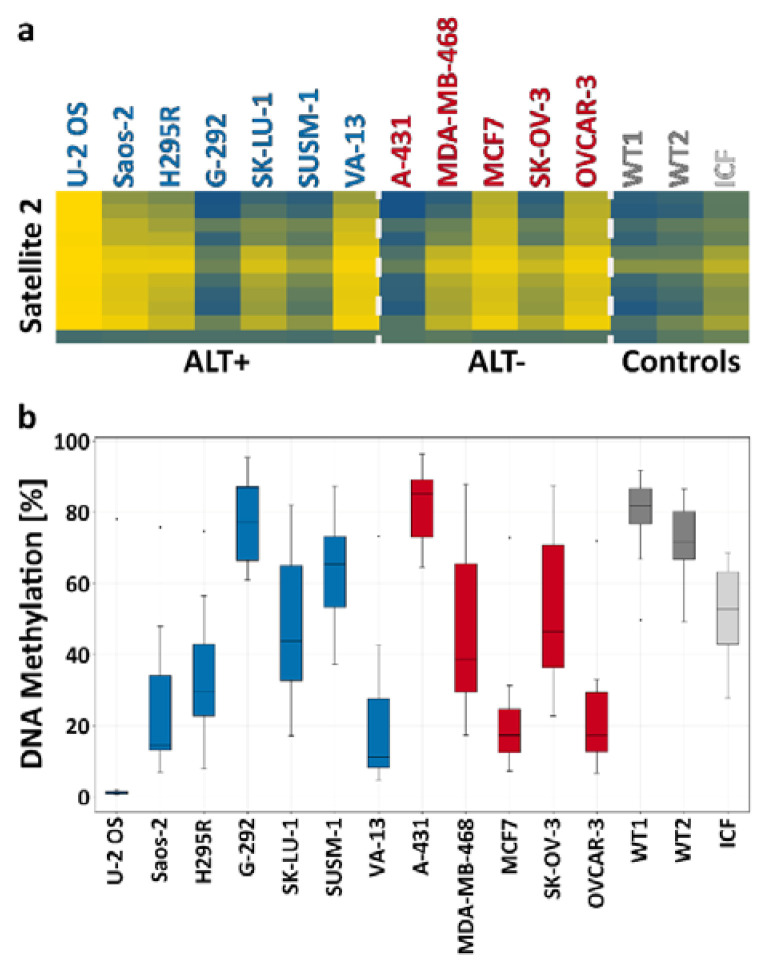
Satellite 2 methylation patterns in ALT+ and ALT- cell lines. Satellite 2 (sat2) methylation was analyzed by high-throughput sequencing of PCR-amplified bisulfite-converted DNA in ALT+ and ALT- cell lines and compared to WT fibroblasts (WT1—UN, WT2—FSE) and ICF1 fibroblast-like cells. (**a**) A heat map illustrating the percentage of methylation across the sat2 amplicons. Each line represents a specific CpG site, in the 5′ to 3′ direction of the sequence (top to bottom). (**b**) A box plot presenting the methylation percentage distribution of all CpG sites of the sat2 amplicon for each sample (ALT+—blue, ALT-—red, WT fibroblasts, and ICF fibroblast-like cells—gray shades).

We next wanted to determine whether ALT+ cell lines are hypomethylated in repetitive regions other than subtelomeres. While one study described hypomethylation of satellite 2 (sat2) and Alu repeats in ALT+ cells [21], another did not find hypomethylation of the NBL2 pericentromeric repeat in ALT+ cells [20]. Here, we inspected the methylation at two pericentromeric repeats, sat2 and NBL1, both hypomethylated in ICF1 syndrome cells [31,47]. We analyzed various ALT+ cell lines and utilized DNA from ICF1 LCLs or sperm as hypomethylated controls, and WT fibroblasts and cord blood DNA as methylated controls. DNA methylation was tested for these repeats by Southern analysis (Figure S3), and sat2 repeats were additionally examined by targeted bisulfite analysis (Figure 3). Both Southern and bisulfite analyses found that sat2 was variably methylated in ALT+ cell lines, starting from an average of 8.2% in U-2 OS and reaching 77.8% in G-292, a value comparable to that found for WT fibroblasts (an average of 75.5%) (Figure 3). The Southern analysis of NBL1 similarly demonstrated hypomethylation to different degrees, depending on the cell line studied (Figure S3b). However, there was no correlation between the degree of hypomethylation at the two examined pericentromeric repeats in each of the ALT+ cell lines (Figure S3c). Likewise, bisulfite analysis of sat2 methylation in ALT- cell lines demonstrated a large variation in average DNA methylation values (22.4-81.6) (Figure 3). In summary, similar to the subtelomeric regions, pericentromeric repeats also displayed varying levels of hypomethylation in cells utilizing the ALT pathway.

### 3.4. TERRA Levels Do Not Allow to Distinguish between ALT+ and ALT- Cell Lines

Although we examined subtelomeric methylation at many chromosome-ends, we could not rule out that members of the remaining unexamined subtelomeres are hypomethylated in all ALT+ cell lines, but not in ALT- cell lines. Subtelomeric hypomethylation is strongly associated with high TERRA expression [14,15,31], and elevated TERRA expression was previously demonstrated in several ALT+ cell lines [18,19,22]. We, therefore, proceeded to ask whether TERRA levels are elevated in all the ALT+ cell lines we studied here, and if they are, whether this is true for TERRA originating from different chromosome ends. TERRA expression was originally studied by Northern analysis, but this analysis can be misleading, as the long telomere lengths in ALT can produce strong hybridization signals even when TERRA levels are low.

**Figure 4:**
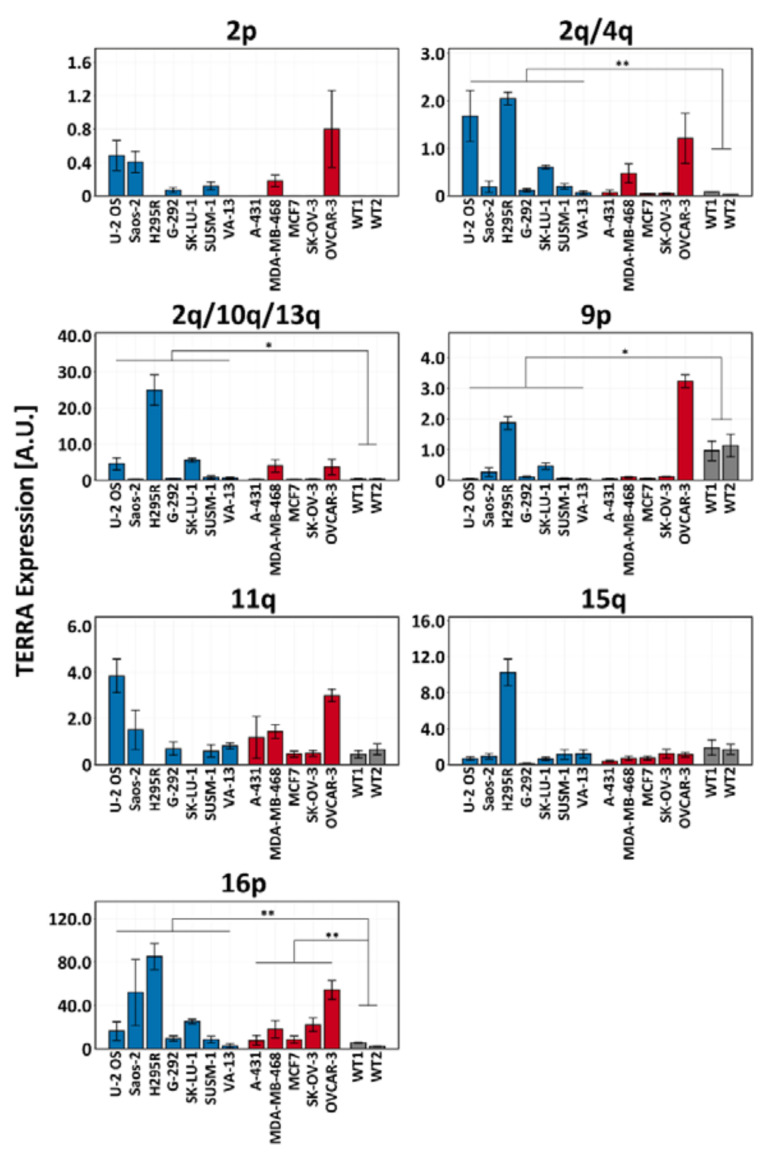
TERRA levels from individual chromosome ends in ALT+ and ALT- cell lines. TERRA expression was studied from seven amplicons representing ten subtelomeres. Values are presented in arbitrary units (A.U.). ALT+ samples appear in blue, ALT- in red, and WT fibroblasts in gray. Bars and error bars depict means and SEM for each sample. Graphs represent at least two experimental repeats. Missing bars, as in the case of several samples in subtelomeres 2p and 11q graphs, indicate a lack of amplification. Expression levels for each amplicon were compared between groups by a two-tailed Welch’s *t*-test (*p* < 0.05 *, *p* < 0.01 **).

To this end, we performed RT-qPCR utilizing subtelomeric-specific primers targeted to regions immediately upstream from the telomeric repeats. We analyzed TERRA originating only at chromosome ends that contain a clear telomeric tract [15]. All TERRA molecules generated from a specific TERRA promoter contain the subtelomeric region positioned between the TERRA transcription start site and the telomeric repeats, but may vary in length in the 3’ region containing the hexameric repeats. Therefore, amplification of the 5’- subtelomeric component of TERRA, which is not affected by the number of telomeric repeats, provides a reliable quantitative measure of TERRA levels. Altogether, we analyzed seven amplicons that represent TERRA produced from 10 chromosome ends (Figure 4) and compared the levels of expression in ALT+ and ALT- cell lines. We also examined TERRA from 5p and 10p/18p chromosome ends but could not detect their expression in any of the cell lines. When comparing TERRA expression between WT fibroblasts and the ALT+ group, we detected significant differences in several of the examined subtelomeres, with average TERRA levels being elevated in the ALT+ cells. However, we could not identify any significant difference in TERRA expression at any of the examined chromosome ends between the ALT+ and ALT- groups, or between the ALT- and the WT groups. In addition, when comparing the samples within the ALT+ group, significant differences were apparent in the expression of most analyzed amplicons (Table S5). Since subtelomeric DNA methylation levels also varied at different chromosome ends (Figure 2), we asked whether an association between TERRA and subtelomeric DNA methylation levels is apparent at any of the regions we assayed for both traits across all or most of the studied cell lines (Figure S4a). While both WT controls displayed similar associations between subtelomeric DNA methylation and TERRA levels, neither the ALT+ nor the ALT- cell lines showed any consistent association at any of the compared chromosome ends.

### 3.5. Subtelomeric H3K9 and H3K4 Trimethylation Vary in ALT+ Cell Lines

**Figure 5:**
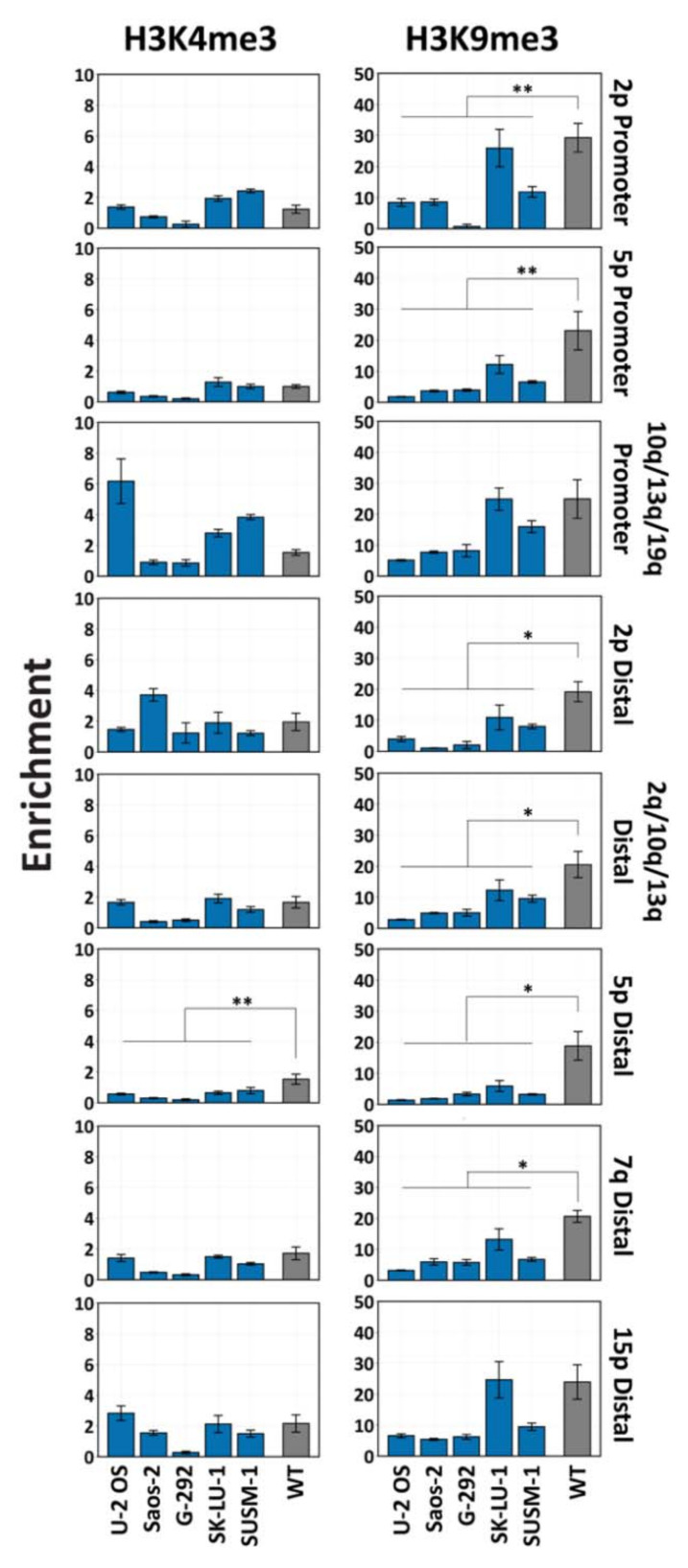
Subtelomeric histone modifications in ALT+ cell lines compared to WT fibroblasts. Enrichment for H3K4me3 (left) and H3K9me3 (right) was determined by ChIP at five TERRA promoter regions and seven distal subtelomeric regions. Enrichment was calculated as the fold increase over the background of a negative control region amplified in the same sample (Hoxa 7 transcription start site for H3K4me3 and *GAPDH* promoter for H3K9me3). Bars and error bars represent means and SEM of at least three experimental repeats. Significant differences within the ALT+ samples (Tables S6 and S7) and between the ALT+ group and WT fibroblasts were tested for by a two-tailed Mann–Whitney U-test (*p* < 0.05 * and *p* < 0.01 **).

Distinct chromatin characteristics at telomeric regions were proposed to underlie ALT-pathway activation [22,48,49]. Previous studies focused on the chromatin packaging of the telomeric repeat region. Here, we directed our analysis to subtelomeric regions that encompass the TERRA promoter regions and studied by ChIP analysis the levels of H3K9 and H3K4 trimethylation at eight chromosome ends in several ALT-pos cells in comparison to WT fibroblasts. Our analyses revealed that, except for SK-LU-1 at certain chromosome ends, H3K9me3 levels are lower in ALT+ cell lines in comparison to WT fibroblasts, suggesting a reduction in the subtelomeric heterochromatic packaging in most ALT+ cells. Significant differences between the group of ALT+ cell lines and WT fibroblasts were apparent at 2p, 5p, 2q/10q/13q, and 7q chromosome ends (Figure 5). However, despite the reduction in H3K9me3 in most ALT+ cells, the levels of H3K4me3 were not significantly elevated in this group of cell lines compared to WT fibroblasts. In the majority of subtelomeres, we found similar enrichment levels of H3K4me3 in the ALT+ compared to the control fibroblasts, with a significantly lower enrichment in the ALT+ group detected only in the case of the 5p-distal region (*p* < 0.01). Moreover, ALT+ samples varied significantly from each other in the enrichment of both H3K9me3 and H3K4me3 modifications at subtelomeres (Tables S6 and S7, respectively). Likewise, no consistent association could be detected between TERRA levels to either H3K4 or H3K9 trimethylation levels at any of the chromosome ends for which we had measured both traits (Figure S4b,c).

## 4. Discussion

Epigenetic changes are a central force in cancer development and a valid target for therapeutic approaches [45,50]. The epigenetic characteristics of telomeric regions in cells utilizing the ALT pathway have been the subject of numerous studies over more than a decade. Nevertheless, there is no consensus regarding the epigenetic traits common to all ALT+ cancers (reviewed in [13,51](. Here, we performed a comprehensive analysis of various epigenetic characteristics of subtelomeric regions to identify traits common to all cells relying on this pathway for telomere maintenance.

In previous publications, elevated levels of TERRA were proposed to play a role in enabling recombination at telomeres in ALT-positive cells [19], and subtelomeric hypomethylation was suggested to enhance TERRA transcription [14,31,43]. Other reports, in contrast, suggested that subtelomeric hypomethylation is not a prerequisite for telomeric recombination in ALT+ cells [18,21]. In the present report, we analyzed numerous subtelomeric regions containing TERRA promoters that reside within 1.5 kb of the telomere tract. This distinguishes the present study from previous studies, where only few subtelomeres were analyzed [18], where methylation was studied collectively for many subtelomeres [22], or where analyzed regions were at a distance of 10 kb or more from the telomere tract [20,21]. Our findings that the catalytically active isoforms of DNMT3B are expressed to a lesser degree in the majority of ALT+ cells in comparison to ALT- cells would have been consistent with subtelomeric hypomethylation in ALT+ cells. However, the targeted bisulfite analysis we conducted did not detect a clear hypomethylated pattern across all ALT+ cells as well as no significant difference between the groups of ALT+ and ALT- cells. Certain subtelomeric regions such as 1q/21q and 2q/4q were hypomethylated in many of the cells of both groups compared to WT-fib, but not in all. Others, such as 4p, were hypermethylated in the majority of cells from both groups, but again with exceptions. In general, we could not detect any region that demonstrated a significant difference between ALT+ and ALT- cells, or a consensus pattern within the group members. The statistical analyses demonstrated that a high similarity in DNA methylation patterns was frequently found between cells of the two opposite groups (Figure 2d). In addition, in contrast to previous reports [18], we were unable to detect global subtelomeric hypermethylation within the group of ALT-/telomerase+ cancer cells. Thus, our analysis of a large group of subtelomeric regions confirms the previous suggestion that ALT+ cells show variable degrees of subtelomeric methylation, and we cannot exclude that this may extend to intra-heterogeneity of subtelomeric DNA methylation patterns within individual cell lines [18]. Nevertheless, even though we studied 14 different chromosome ends, we cannot exclude that other TERRA promoter regions that were not sampled in this study may show an ALT-specific hypomethylated signature. We can conclude that at the least there is no general and significant trend of subtelomeric DNA hypomethylation in ALT+ distal subtelomeric regions. This conclusion appears to also be true for other repetitive regions of the genome. Our DNA methylation analyses of satellite 2 by targeted bisulfite sequencing and by Southern blotting (Figure 3 and Figure S3a) also demonstrate an extreme variation in DNA methylation, from exceptionally low levels in U-2 OS to high levels in G-292. It should be noted that, at least for satellite 2 repeats, noticeable variation in DNA methylation levels was also evident in the group of ALT- cancer cells.

The variation between samples in both ALT+ and ALT- groups extended also to TERRA expression levels. Our RT-qPCR analysis of the most distal subtelomeric regions from nine chromosome ends did not reveal a common expression pattern of any of the analyzed TERRAs within either of the two groups (Figure 4). Additionally, we found no significant difference in TERRA levels between both groups, and while TERRA expression was higher at several chromosome ends compared to WT fibroblasts, this was true for both the ALT+ and ALT- groups. Certain ALT+ cell lines tended to express more TERRA from many chromosome ends, such as U-2 OS and H295R. However, when studying a large repertoire of cell lines it is clear that this is not a common characteristic of all ALT+ cells. As with subtelomeric DNA methylation, we cannot exclude that TERRA is transcribed in ALT+ cells from one or several chromosome ends that were not examined in this study. As TERRA was also recently demonstrated to localize to telomeres in *trans* [52], one or few hyperactive TERRA promoters could conceivably generate sufficient TERRA required for activating telomeric recombination at many chromosome ends. However, it should be noted that certain ALT- cells, such as OVCAR-3, also highly express TERRA from many chromosome ends, suggesting that additional factors, besides its cellular abundance, are required for TERRA to promote telomeric recombination.

The controversy regarding the epigenetic characteristics of ALT+ telomeres extends to the histone modifications and chromatin compaction at ALT telomeres [53]. Telomere packaging as heterochromatin was considered essential for suppressing ALT [22,54]; however, recent studies support the enrichment of H3K9me3 at ALT telomeres, indicating that a shift to heterochromatic characteristics is a hallmark of the ALT pathway [48,49]. Subtelomeric chromatin is not necessarily identical to telomeric chromatin, and a specific histone modification in ALT+ cells may play a different role at subtelomeres compared to telomeric repeats (reviewed in [55,56]). In this study, we considered the potential role for TERRA in ALT activation and, therefore, concentrated on determining whether distal subtelomeric regions encompassing TERRA promoters exhibit distinct H3K4me3 and H3K9me3 patterns in ALT+ cells compared to WT fibroblasts. We found that for the five ALT+ cells we studied, H3K9 trimethylation was reduced in most subtelomeric chromatin in comparison to WT fibroblasts. Thus, at least within the most distal subtelomeric regions the chromatin does not acquire clear heterochromatic features. On the other hand, we did not observe in ALT+ subtelomeric regions enrichment of H3K4me3, a modification associated with enhanced transcriptional activity, and this correlated with the lack of elevated TERRA transcription at most chromosome ends in any of the members of the ALT+ group.

Deciphering the factors that drive the ALT pathway is essential for designing therapy for tumors that maintain telomeres in this manner. Epigenetic changes at telomeric regions were shown to function as one of these crucial factors, yet when we focused on subtelomeres, our analyses could not define any characteristic common to all or even the majority of ALT+ cells. The wide variability in the epigenetic traits of these regions could potentially stem from the differential tissue source of the studied cell lines. However, we could not detect any similarities even among cell lines derived from an identical source, such as U-2 OS, Soas-2, and G-292, all osteosarcomas. An additional factor that could hypothetically influence epigenetic traits is the mechanism by which ALT was activated. However, at least when studying subtelomeric DNA methylation, no similarities were detected among the five ALT+ cell lines derived from tumors in comparison to the two ALT+ cell lines, SUSM-1 and VA-13, that were immortalized by chemical means or by SV40 large T-antigen. Thus, while we cannot conclusively exclude that certain epigenetic characteristics at telomeric and subtelomeric regions are crucial for the ALT pathway, the majority may reflect the outcome of stochastic processes that occur during cellular immortalization. The high variability uncovered between ALT+ samples both in this study, as well as in previous reports, could also support the notion previously suggested [8] that the assemblage of these tumors cannot be treated as a uniform group and that more than one mechanism may pave the way to telomere maintenance by recombination. Future studies of tumors utilizing the ALT pathway are necessary to determine whether the subtelomeric and telomeric epigenetic variability observed in ALT+ cell lines are similarly observed in tumors in vivo.

## Figures and Tables

**Table 1:** ALT+ and ALT- Cell Lines Utilized in This Study.

Cell Type	Telomere Maintenance by:	Source	ATCC# or Reference
**Saos-2**	ALT	Osteosarcoma	ATCC HTB-85
**U-2 OS**	ALT	Osteosarcoma	ATCC HTB-96
**G-292**	ALT	Osteosarcoma	ATCC CRL-1423
**SK-LU-1**	ALT	Lung adenocarcinoma	ATCC HTB-57
**H295R**	ALT	Adrenal carcinoma	ATCC CRL-2128
**SUSM-1**	ALT	Chemically transformed human liver fibroblasts	[29,30]
**VA-13**	ALT	Lung fibroblasts immortalized by SV-40 large T antigen	ATCC CCL-75.1
**A-431**	telomerase	Epidermoid carcinoma	ATCC CRL-1555
**MCF7**	telomerase	Breast adenocarcinoma	ATCC HTB-22
**MDA-MB-468**	telomerase	Breast adenocarcinoma	ATCC HTB-132
**OVCAR-3**	telomerase	Ovarian adenocarcinoma	ATCC HTB-161
**SK-OV-3**	telomerase	Ovarian adenocarcinoma	ATCC HTB-77

## Data Availability

Data is contained within the article or supplementary material.

## Supplementary Materials

The following are available online at https://www.mdpi.com/article/10.3390/life11040278/s1, Figure S1: Telomere length analysis of ALT+ and ALT- cell lines, Figure S2: Subtelomeric methylation analysis in ALT+ and ALT- cells by Southern analyses, Figure S3: Southern analysis of DNA methylation of pericentromeric repeats in ALT+ lines, Figure S4: Association of TERRA expression with subtelomeric DNA methylation in ALT+ and ALT- cell lines and with histone modifications in ALT+ cell lines, Table S1: Primer sequences for RT-qPCR of *DNMT3B* and *TET* protein family, Table S2: Primer sequences for ChIP and TERRA RT-qPCR analyses, Table S3: *P* values of two-tailed Mann–Whitney U-test comparing *DNMT3B* expression in ALT+ and ALT- samples, Table S4: *P* values of a two-tailed Mann–Whitney U-test comparing *TET* genes expression of ALT+ and ALT- samples, Table S5: *P* values of a two-tailed Welch’s *t*-test comparing TERRA expression levels in ALT+ and ALT- samples, Table S6: *P* values of a two-tailed Mann–Whitney U-test comparing H3K9me3 levels in ALT+ samples and FSE WT cells, Table S7: *P* values of a two-tailed Mann–Whitney U-test comparing H3K4me3 levels of ALT+ samples and FSE WT cells.
