# Supplementary material for: Epigenetic Characteristics of Human Subtelomeres Vary in Cells Utilizing the Alternative Lengthening of Telomeres (ALT) Pathway
Source: Life (Basel). 2021 Mar 26;11(4):278. doi: 10.3390/life11040278 (PMC8065733; doi:10.3390/life11040278)
Supplement: Supplementary file 1 [file life-11-00278-s001.pdf]

life

MDPI

Supplementary Materials of Epigenetic Characteristics of Human Subtelomeres Vary in Cells Utilizing the Alternative Lengthening of Telomeres (ALT) Pathway

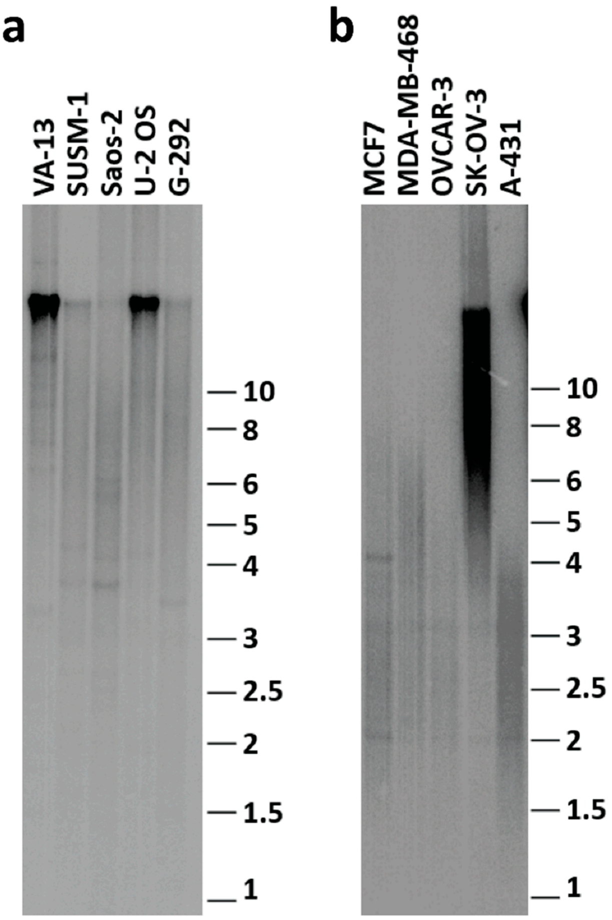

Figure S1. Telomere length analysis of ALT+ and ALT- cell lines. Terminal Restriction Fragment (TRF) analysis was performed on DNA extracted from (**a**) ALT+ cell lines, and (**b**) ALT- cell lines. Size markers in kilobases appear to the right.

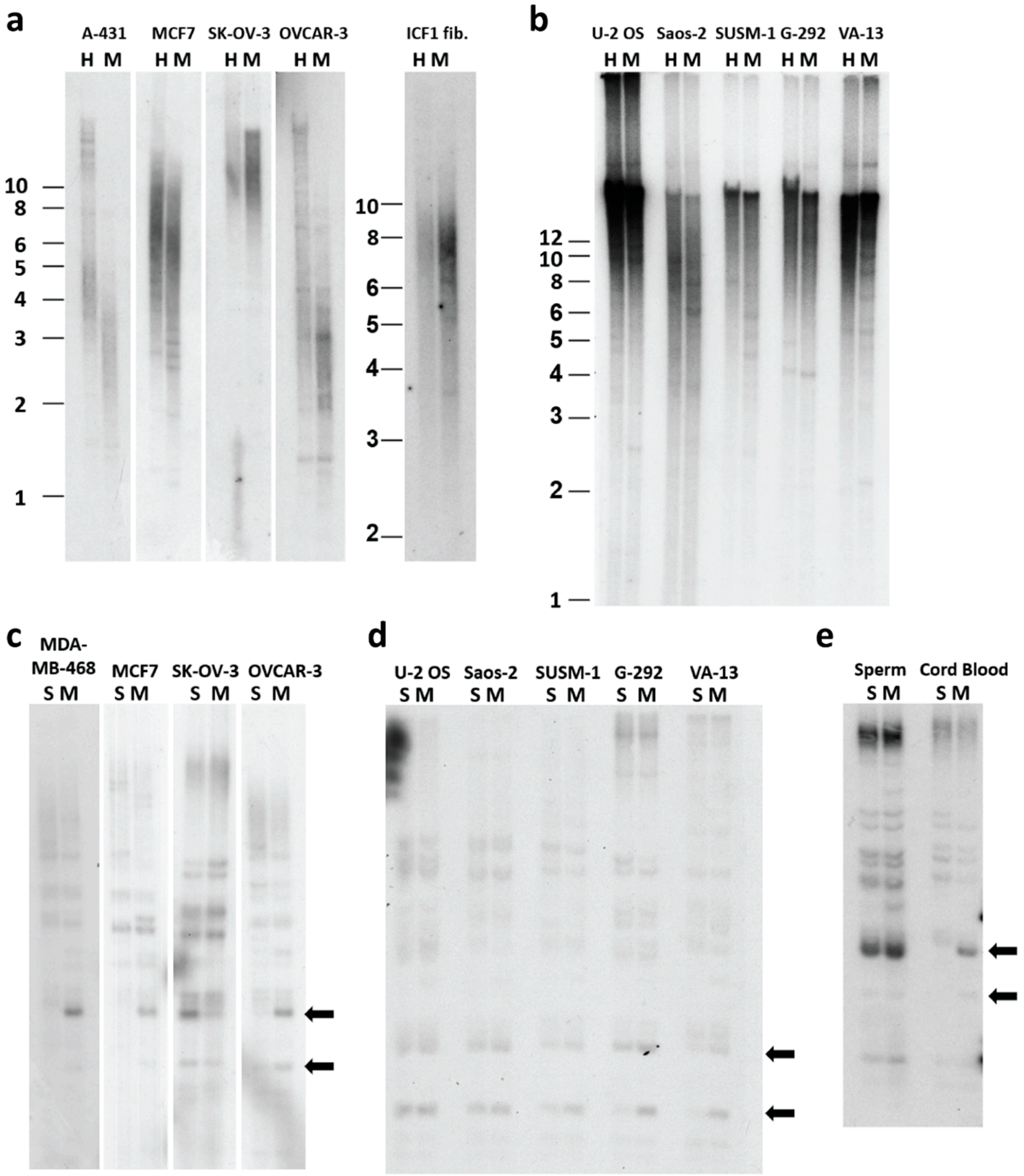

Figure S2. Subtelomeric methylation Southern analysis in ALT+ and ALT- cell lines. Terminal Restriction Fragment (TRF) analysis of (**a**) four ALT- cell lines and a control hypomethylated ICF1 LCL and (**b**) five ALT+ cell lines. Samples were digested with either *HpaII* (H) or *MspI* (M). Size markers in kilobases appear to the left. (**c**-**e**) Methylation analysis with the subtelomeric Hutel probe. Samples were digested with either *Sau3AI* (S) or *MboI* (M). Arrows point to hybridization bands that appear following the *Sau3AI* digestion only when the hybridized region is hypomethylated. (**c**) Analysis of four ALT- cell lines. (**d**) Analysis of five ALT+ cell lines. (**e**) Analysis of a hypomethylated DNA control (sperm DNA) and a methylated control (cord blood DNA).

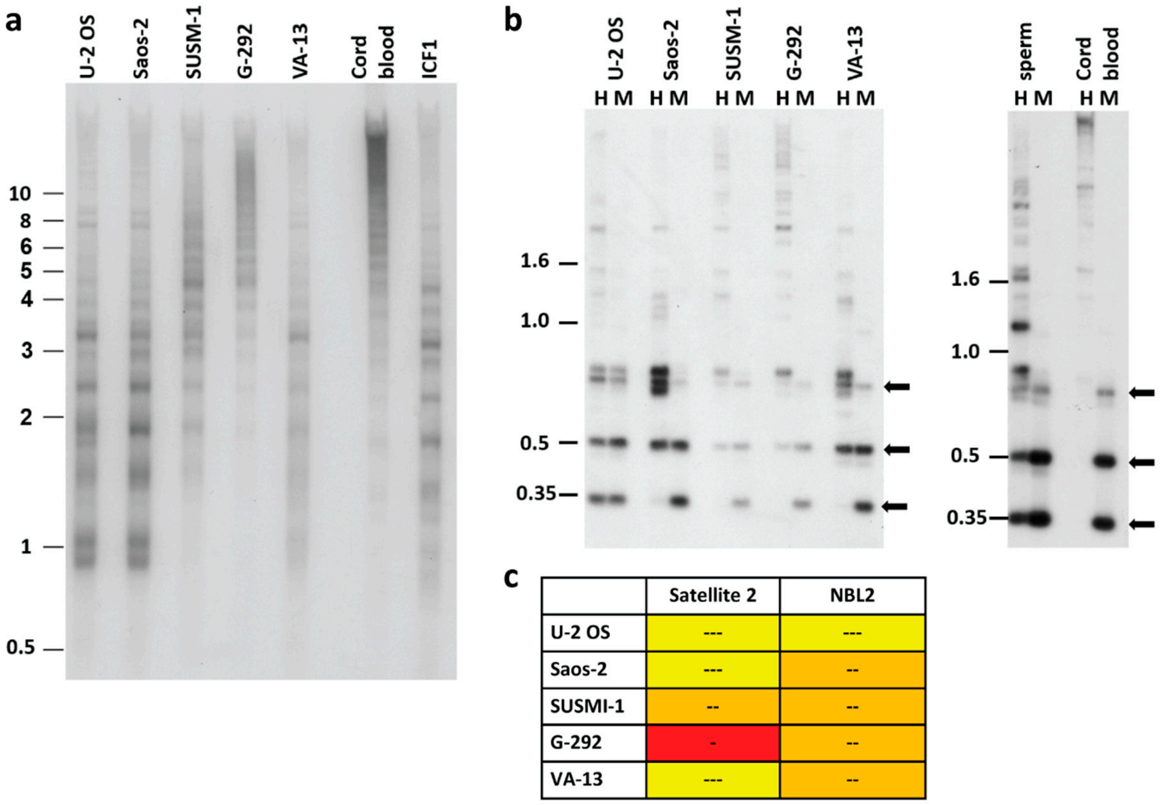

c

	Satellite 2	NBL2
U-2 OS	---	---
Saos-2	---	--
SUSMI-1	--	--
G-292	-	--
VA-13	---	--

Figure S3. Southern analysis of DNA methylation of pericentromeric repeats in ALT+ cell lines**.** (**a**) Methylation analysis of satellite 2 repeats in five ALT+ cell lines. DNA samples were digested with the methylation sensitive *BstBI* restriction enzyme. Cord blood DNA served as a methylated control and ICF1 LCL DNA as a hypomethylated control. Size markers in kilobases appear to the left. (**b**) Methylation analysis of NBL1 repeats in five ALT+ cell lines. Cord blood DNA served as a methylated control and sperm DNA as a hypomethylated control. Samples were digested with either *HpaII* (H) or *MspI* (M). Arrows point to hybridization bands that appear following the *HpaII* digestion only when the hybridized region is hypomethylated. Size markers in kilobases appear to the left. (c) Comparison of the degree of hypomethylation at both pericentromeric repeats in the ALT+ cell lines. Yellow (---) – hypomethylated, orange (--) – partially hypomethylated, red (-) –methylated.

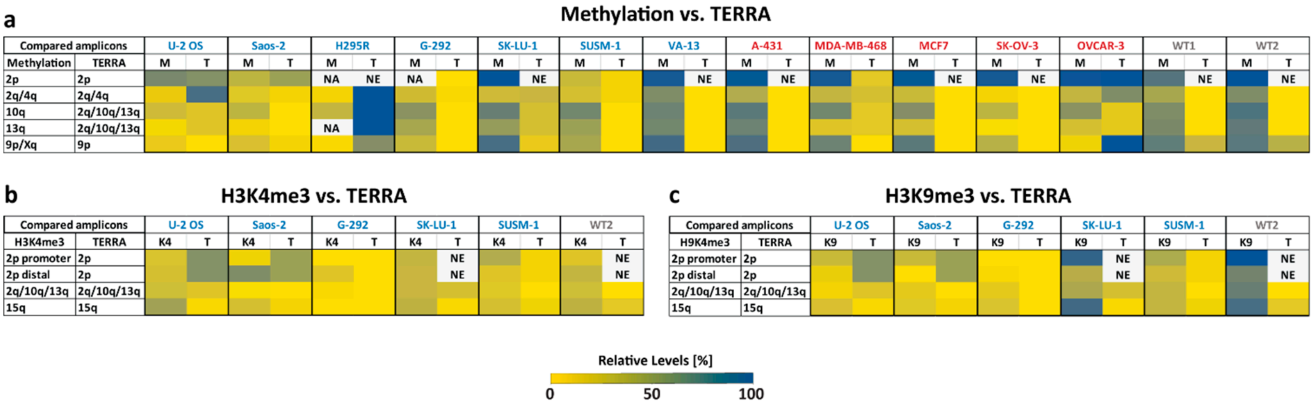

Figure S4. Association of TERRA expression with subtelomeric DNA methylation in ALT+ and ALT- cell lines and with histone modifications in ALT+ cell lines. Comparison of TERRA levels with DNA methylation levels (**a**) and histone H3K4 and H3K9 trimethylation levels (b and c, respectively) at different subtelomeres within each sample. Mean values on the scale of 0–100% were calculated for each of the studied characteristics, for several subtelomeres in each sample. For DNA methylation we calculated the mean percentage of all the CpGs within each amplicon while using the values that were obtained through targeted bisulfite sequencing. For TERRA levels, each subtelomere was adjusted separately since expression levels cannot be compared between different amplicons. The highest value obtained for each subtelomere was set to 100%, and the values of the remaining samples for the same subtelomere were normalized between 0-100% while maintaining the original ratio of expression values between the samples. For histone modifications levels, relative

enrichment values for all subtelomeres within a sample were obtained by calculating the fold increase above the background of the negative control. To obtain mean values on the scale of 0–100% we set the highest value among all the subtelomeres of all samples to 100% for each of the modifications separately, and normalized the remaining values appropriately, while preserving the original ratios between the samples. The colors in the heatmaps depict the adjusted mean values based on the color scale below the maps. NA—Not Available (primers do not amplify the region in the specific sample). NE—Not Expressed. M—DNA methylation, T—TERRA, K4—H3K4 trimethylation, K9—H3K9 trimethylation. ALT+ samples appear in blue, ALT- samples appear in red.

Table S1. Primer sequences for RT-qPCR of *DNMT3B* and *TET* protein family.

Primer	Sequence
DNMT3B-all isoforms-For	5′-CTGTTCAGCCAGCACTTTAATTTG-3′
DNMT3B-all isoforms-Rev	5′-CACCCTAGCTTTCTCCAGAGCAT-3′
DNMT3B-catalytic isoforms-For	5′-GAACAGGCCCGTGATAGCAT-3′
DNMT3B-catalytic isoforms-Rev	5′-TGTACTTTCTTTAACTTGGCTATCCTATTG-3′
TET1-For	5′-GCTGCTGTCAGGGAAATCAT-3′
TET1-Rev	5′-ACCATCACAGCAGTTGGACA-3′
TET2-For	5′-CCAATAGGACAATGATCCAGG-3′
TET2-Rev	5′-TCTGGATGAGCTCTCTCAGG-3′
TET3-For	5′-TCGGAGACACCCTCTACCAG-3′
TET3-Rev	5′-TCGGAGACACCCTCTACCAG-3′
HPRT-For	5′-CTGAGGATTTGGAAAGGGTGTT-3′
HPRT-Rev	5′-AAAGAATTTATAGCCCCCCTTGAG-3′

Table S2. Primer sequences for ChIP and TERRA RT-qPCR analyses.

Primer	Sequence	Used for
Subtelomere 2p promoter-For	5′-GTGGAACCTCAATAATCCGAAAA-3′	ChIP
Subtelomere 2p promoter-Rev	5′-GGACACCACTGTAAGCAAGATAGC-3′	
Subtelomere 5p promoter-For	5′-TGGCACAGCATCGTAGACAAG-3′	ChIP
Subtelomere 5p promoter-Rev	5′-TGGAACCTCAGCAATCTGAAAA-3′	
Subtelomere 10q/13q/19q promoter-For	5′-GGCGCTGGACACCACTGTA-3′	ChIP
Subtelomere 10q/13q/19q promoter-Rev	5′-TGAGTAATCTGAAAAGCCCGTTT-3′	
Subtelomere 7q-For	5′-TTCAGACGGGCTTTTGGTTT-3′	ChIP
Subtelomere 7q-Rev	5′-ATGGTGAATACAATCCTTTCTGTTTG-3′	
Subtelomere 2p-For	5′-CGCATCGACGGTGAATAAAA-3′	ChIP and RT-qPCR
Subtelomere 2p-Rev	5′-GCCTAACTCGTGTCTGACTTTGAG-3′	
Subtelomere 5p-For	5′-GAGTGCATTAGCATACAGGT-3′	ChIP and RT-qPCR
Subtelomere 5p-Rev	5′-TCCTAATGCACACGTAACAC-3′	
Subtelomere 2q/10q/13q-For	5′-AACCTGAACCCTAACCCTCC-3′	ChIP and RT-qPCR
Subtelomere 2q/10q/13q-Rev	5′-ATTGCAGGGTTCAAGTGCAG-3′	
Subtelomere 15q-For	5′-AACCCTAACCACATGAGCAACG-3′	ChIP and RT-qPCR
Subtelomere 15q-Rev	5′-CTCGCCTTAGCTTGGGAG-3′	
Subtelomere 11q-For	5′-CTGATTATTCAGGGCTGCAAA-3′	RT-qPCR
Subtelomere 11q-Rev	5′-GCCGCATCGACGGTGAATAA-3′	
Subtelomere 9p-For	5′-GGGCGCATTAACGGTGAATA-3′	RT-qPCR
Subtelomere 9p-Rev	5′-CCGCACTGAACCGCTCTAAC-3′	
Subtelomere 2q/4q-For	5′-GGTGAATAAAATCTTTCCTGTTTGC-3′	RT-qPCR
Subtelomere 2q/4q-Rev	5′-TTTTCGTTTCCCGCTTTCC-3′	
Subtelomere 16p-For	5′-AACGGTTCAGTGTGGAAAATGG-3′	RT-qPCR
Subtelomere 16p-Rev	5′-CAACTGGACCCTGCAATGC-3′	
Subtelomere 10p/18p-For	5′-CCTTCTAACTGGACTCTGAC-3′	RT-qPCR
Subtelomere 10p/18p-Rev	5′-GCCACAGCGACGGTAAATAA-3′	
β-actin-For	5′-TGTACGCCAACACAGTGCTG-3′	RT-qPCR
β-actin-Rev	5′-GCTGGAAGGTGGACAGCGA-3′	
Hoxa 7 TSS-For	5′- TACTCACCTCCCCTTTCCCA-3′	ChIP
Hoxa 7 TSS-Rev	5′- GTACCCTGCTCAGCTCCATC-3′	
GAPDH promoter-For	5′-AAAGCCCGCACCAACCAT-3′	ChIP
GAPDH promoter-Rev	5′-AGTCCCTGACCCTGCCTTTC-3′	

Table 3. P values of two-tailed Mann–Whitney U-test comparing *DNMT3B* expression in ALT+ and ALT- samples.

DNMT3B-Catalytically Active Isoforms	U-2 OS	Saos-2	H295R	G-292	SK-LU-1	SUSM-1	VA-13	A-431	MDA-MB-468	MCF7	SK-OV-3	OVCAR-3
U-2 OS	1	0.030383	0.05183	0.030383	0.05183	0.030383	0.030383	0.111612	0.470486422	0.060602	0.030383	0.030383
Saos-2	0.030383	1	0.595883	0.885234	0.595883	0.030383	0.112351	0.05183	0.030382822	0.030383	0.030383	0.030383
H295R	0.05183	0.595883	1	0.859684	0.662521	0.111612	0.215925	0.080856	0.051829927	0.05183	0.05183	0.05183
G-292	0.030383	0.885234	0.859684	1	0.859684	0.060602	0.193931	0.05183	0.030382822	0.030383	0.060602	0.030383
SK-LU-1	0.05183	0.595883	0.662521	0.859684	1	0.111612	0.215925	0.080856	0.051829927	0.05183	0.05183	0.05183
SUSM-1	0.030383	0.030383	0.111612	0.060602	0.111612	1	0.470486	0.05183	0.030382822	0.030383	0.030383	0.030383
VA-13	0.030383	0.112351	0.215925	0.193931	0.215925	0.470486	1	0.05183	0.030382822	0.030383	0.030383	0.030383
A-431	0.111612	0.05183	0.080856	0.05183	0.080856	0.05183	0.05183	1	0.376759118	0.05183	0.215925	0.05183
MDA-MB-468	0.470486	0.030383	0.05183	0.030383	0.05183	0.030383	0.030383	0.376759	1	0.060602	0.112351	0.030383
MCF7	0.060602	0.030383	0.05183	0.030383	0.05183	0.030383	0.030383	0.05183	0.06060197	1	0.030383	0.470486
SK-OV-3	0.030383	0.030383	0.05183	0.060602	0.05183	0.030383	0.030383	0.215925	0.112351198	0.030383	1	0.030383
OVCAR-3	0.030383	0.030383	0.05183	0.030383	0.05183	0.030383	0.030383	0.05183	0.030382822	0.470486	0.030383	1
**DNMT3 -All Isoforms**	**U-2 OS**	**Saos-2**	**H295R**	**G-292**	**SK-LU-1**	**SUSM-1**	**VA-13**	**A-431**	**MDA-MB-468**	**MCF7**	**SK-OV-3**	**OVCAR-3**
U-2 OS	1	0.060602	0.030383	0.112351	0.060602	0.030383	0.030383	0.111612	0.193930852	0.312321	0.193931	0.312321
Saos-2	0.060602	1	0.470486	0.885234	0.665006	0.193931	0.312321	0.859684	0.665005542	0.312321	0.665006	0.193931
H295R	0.030383	0.470486	1	0.470486	0.665006	0.665006	0.665006	0.376759	0.193930852	0.060602	0.193931	0.112351
G-292	0.112351	0.885234	0.470486	1	0.665006	0.193931	0.312321	0.859684	0.885233914	0.312321	0.885234	0.312321
SK-LU-1	0.060602	0.665006	0.665006	0.665006	1	0.470486	0.470486	0.859684	0.665005542	0.312321	0.470486	0.193931
SUSM-1	0.030383	0.193931	0.665006	0.193931	0.470486	1	0.885234	0.376759	0.193930852	0.060602	0.193931	0.030383
VA-13	0.030383	0.312321	0.665006	0.312321	0.470486	0.885234	1	0.215925	0.06060197	0.060602	0.060602	0.030383
A-431	0.111612	0.859684	0.376759	0.859684	0.859684	0.376759	0.215925	1	0.595883091	0.215925	0.376759	0.215925
MDA-MB-468	0.193931	0.665006	0.193931	0.885234	0.665006	0.193931	0.060602	0.595883	1	0.665006	0.885234	0.312321
MCF7	0.312321	0.312321	0.060602	0.312321	0.312321	0.060602	0.060602	0.215925	0.665005542	1	0.665006	0.665006
SK-OV-3	0.193931	0.665006	0.193931	0.885234	0.470486	0.193931	0.060602	0.376759	0.885233914	0.665006	1	0.312321

OVCAR-3	0.312321	0.193931	0.112351	0.312321	0.193931	0.030383	0.030383	0.215925	0.312321422	0.665006	0.312321	1

ALT+ samples are marked in blue; ALT- samples are marked in red. The values in the cells represent the P value of the two compared samples. Significant values (P < 0.05) are marked in yellow.

Table S4. P values of a two-tailed Mann–Whitney U-test comparing *TET* genes expression of ALT+ and ALT- samples.

TET1	U-2 OS	Saos-2	H295R	G-292	SK-LU-1	SUSM-1	VA-13	A-431	MDA-MB-468	MCF7	SK-OV-3	OVCAR-3
U-2 OS	1	0.382733	0.595883	0.382733	0.05183	0.382733	0.080856	0.382733	0.662520584	0.080856	0.19043	1
Saos-2	0.382733	1	0.859684	0.382733	0.05183	0.662521	0.080856	0.382733	0.382733089	0.080856	0.19043	0.662521
H295R	0.595883	0.859684	1	0.376759	0.030383	0.859684	0.05183	0.859684	0.111611768	0.05183	0.215925	0.595883
G-292	0.382733	0.382733	0.376759	1	0.111612	0.662521	0.19043	1	0.190430264	0.382733	0.662521	0.382733
SK-LU-1	0.05183	0.05183	0.030383	0.111612	1	0.111612	0.859684	0.111612	0.051829927	0.595883	0.111612	0.111612
SUSM-1	0.382733	0.662521	0.859684	0.662521	0.111612	1	0.19043	0.662521	0.190430264	0.382733	0.382733	0.382733
VA-13	0.080856	0.080856	0.05183	0.19043	0.859684	0.19043	1	0.19043	0.080855598	0.662521	0.19043	0.080856
A-431	0.382733	0.382733	0.859684	1	0.111612	0.662521	0.19043	1	0.190430264	0.382733	1	0.382733
MDA-MB-468	0.662521	0.382733	0.111612	0.19043	0.05183	0.19043	0.080856	0.19043	1	0.080856	0.080856	0.662521
MCF7	0.080856	0.080856	0.05183	0.382733	0.595883	0.382733	0.662521	0.382733	0.080855598	1	0.382733	0.080856
SK-OV-3	0.19043	0.19043	0.215925	0.662521	0.111612	0.382733	0.19043	1	0.080855598	0.382733	1	0.382733
OVCAR-3	1	0.662521	0.595883	0.382733	0.111612	0.382733	0.080856	0.382733	0.662520584	0.080856	0.382733	1
**TET2**	**U-2 OS**	**Saos-2**	**H295R**	**G-292**	**SK-LU-1**	**SUSM-1**	**VA-13**	**A-431**	**MDA-MB-468**	**MCF7**	**SK-OV-3**	**OVCAR-3**
U-2 OS	1	0.662521	0.05183	0.19043	0.111612	0.19043	0.080856	0.080856	1	0.386476	0.19043	1
Saos-2	0.662521	1	0.05183	0.080856	0.05183	0.080856	0.080856	0.080856	0.662520584	0.77283	0.080856	0.662521
H295R	0.05183	0.05183	1	0.595883	0.885234	0.595883	0.215925	0.215925	0.051829927	0.105193	0.859684	0.111612
G-292	0.19043	0.080856	0.595883	1	0.595883	0.382733	1	1	0.382733089	0.148915	1	0.19043
SK-LU-1	0.111612	0.05183	0.885234	0.595883	1	0.376759	0.595883	0.859684	0.051829927	0.105193	0.859684	0.111612
SUSM-1	0.19043	0.080856	0.595883	0.382733	0.376759	1	0.080856	0.19043	0.382733089	0.148915	0.662521	0.19043
VA-13	0.080856	0.080856	0.215925	1	0.595883	0.080856	1	0.662521	0.080855598	0.148915	0.382733	0.080856
A-431	0.080856	0.080856	0.215925	1	0.859684	0.19043	0.662521	1	0.080855598	0.148915	0.382733	0.080856

**MDA-MB-468**	1	0.662521	0.05183	0.382733	0.05183	0.382733	0.080856	0.080856	1	0.77283	0.080856	1
**MCF7**	0.386476	0.77283	0.105193	0.148915	0.105193	0.148915	0.148915	0.148915	0.772829993	1	0.148915	0.386476
**SK-OV-3**	0.19043	0.080856	0.859684	1	0.859684	0.662521	0.382733	0.382733	0.080855598	0.148915	1	0.19043
**OVCAR-3**	1	0.662521	0.111612	0.19043	0.111612	0.19043	0.080856	0.080856	1	0.386476	0.19043	1

**TET3**	**U-2 OS**	**Saos-2**	**H295R**	**G-292**	**SK-LU-1**	**SUSM-1**	**VA-13**	**A-431**	**MDA-MB-468**	**MCF7**	**SK-OV-3**	**OVCAR-3**
**U-2 OS**	1	0.662521	0.662521	0.080856	0.19043	0.080856	0.080856	0.382733	0.772829993	0.77283	0.19043	1
**Saos-2**	0.662521	1	1	0.19043	0.662521	0.080856	0.19043	0.662521	0.772829993	0.77283	0.662521	0.382733
**H295R**	0.662521	1	1	0.382733	0.662521	0.19043	0.382733	0.662521	0.386476231	0.77283	0.662521	0.382733
**G-292**	0.080856	0.19043	0.382733	1	0.662521	0.382733	0.662521	0.382733	0.148914673	0.148915	0.382733	0.19043
**SK-LU-1**	0.19043	0.662521	0.662521	0.662521	1	0.662521	0.662521	0.662521	0.386476231	0.386476	1	0.382733
**SUSM-1**	0.080856	0.080856	0.19043	0.382733	0.662521	1	1	0.382733	0.148914673	0.148915	0.382733	0.080856
**VA-13**	0.080856	0.19043	0.382733	0.662521	0.662521	1	1	0.382733	0.148914673	0.148915	0.382733	0.080856
**A-431**	0.382733	0.662521	0.662521	0.382733	0.662521	0.382733	0.382733	1	0.386476231	0.386476	1	0.382733
**MDA-MB-468**	0.77283	0.77283	0.386476	0.148915	0.386476	0.148915	0.148915	0.386476	1	0.698535	0.386476	0.77283
**MCF7**	0.77283	0.77283	0.77283	0.148915	0.386476	0.148915	0.148915	0.386476	0.698535358	1	0.77283	0.77283
**SK-OV-3**	0.19043	0.662521	0.662521	0.382733	1	0.382733	0.382733	1	0.386476231	0.77283	1	0.382733
**OVCAR-3**	1	0.382733	0.382733	0.19043	0.382733	0.080856	0.080856	0.382733	0.772829993	0.77283	0.382733	1

ALT+ samples are marked in blue; ALT- samples are marked in red. The values in the cells represent the P value of the two compared samples. Significant values ($P < 0.05$) are marked in yellow.

Table S5. P values of a two-tailed Welch's *t*-test comparing TERRA expression levels in ALT+ and ALT- samples.

Subtelomere 2p	U-2 OS	Saos-2	G-292	SUSM-1	MDA-MB-468	OVCAR-3
U-2 OS	1	0.737746	0.141069	0.171515	0.226982	0.615509
Saos-2	0.737746	1	0.105631	0.135948	0.214615	0.544479
G-292	0.141069	0.105631	1	0.41862	0.31883	0.355669
SUSM-1	0.171515	0.135948	0.41862	1	0.547886	0.37641
MDA-MB-468	0.226982	0.214615	0.31883	0.547886	1	0.402691
OVCAR-3		0.544479	0.355669	0.37641	0.402691	1

Subtelomeres 2q4q	U-2 OS	Saos-2	H295R	G-292	SK-LU-1	SUSM-1	VA-13	A-431	MDA-MB-468	MCF7	SK-OV-3	OVCAR-3
U-2 OS	1	0.10166	0.571364	0.098886	0.180775	0.107719	0.094259	0.093584	0.140092	0.092244	0.092791	0.564469
Saos-2	0.10166	1	0.000494	0.599938	0.064239	0.944277	0.428514	0.439808	0.306446	0.351868	0.368703	0.190154
H295R	0.571364	0.000494	1	0.002891	0.004823	0.00144	0.002755	0.001769	0.004382	0.004137	0.00391	0.252805
G-292	0.098886	0.599938	0.002891	1	0.000796	0.291283	0.402323	0.555639	0.211456	0.166658	0.188741	0.17483
SK-LU-1	0.180775	0.064239	0.004823	0.000796	1	0.007223	0.000575	0.011373	0.57294	0.003932	0.002237	0.37207
SUSM-1	0.107719	0.944277	0.00144	0.291283	0.007223	1	0.147153	0.200397	0.304525	0.117293	0.120941	0.196505
VA-13	0.094259	0.428514	0.002755	0.402323	0.000575	0.147153	1	0.992164	0.177648	0.523786	0.634217	0.164608
A-431	0.093584	0.439808	0.001769	0.555639	0.011373	0.200397	0.992164	1	0.175395	0.69725	0.763982	0.164113
MDA-MB-468	0.140092	0.306446	0.004382	0.211456	0.57294	0.304525	0.177648	0.175395	1	0.164333	0.167696	0.297635
MCF7	0.092244	0.351868	0.004137	0.166658	0.003932	0.117293	0.523786	0.69725	0.164333	1	0.71648	0.159512
SK-OV-3	0.092791	0.368703	0.00391	0.188741	0.002237	0.120941	0.634217	0.763982	0.167696	0.71648	1	0.160799
OVCAR-3	0.564469	0.190154	0.252805	0.17483	0.37207	0.196505	0.164608	0.164113	0.297635	0.159512	0.160799	1

Subtelomeres 2q10q13q	U-2 OS	Saos-2	H295R	G-292	SK-LU-1	SUSM-1	VA-13	A-431	MDA-MB-468	MCF7	SK-OV-3	OVCAR-3
U-2 OS	1	0.124795	0.026754	0.13603	0.608671	0.149895	0.140477	0.124229	0.834236	0.124419	0.126848	0.780448
Saos-2	0.124795	1	0.027572	0.114843	0.008247	0.335537	0.368207	0.939708	0.157308	0.929278	0.735374	0.348156
H295R	0.026754	0.027572	1	0.028065	0.042037	0.027669	0.027819	0.027541	0.024946	0.027563	0.027667	0.023656
G-292	0.13603	0.114843	0.028065	1	0.00942	0.518426	0.681071	0.124656	0.1726	0.066447	0.165933	0.36712

**SK-LU-1**	0.608671	0.008247	0.042037	0.00942	1	0.002572	0.002683	0.007937	0.458817	0.008587	0.008507	0.532027
**SUSM-1**	0.149895	0.335537	0.027669	0.518426	0.002572	1	0.739647	0.328949	0.196325	0.329076	0.364105	0.394676
**VA-13**	0.140477	0.368207	0.027819	0.681071	0.002683	0.739647	1	0.358266	0.181082	0.357365	0.414241	0.377482
**A-431**	0.124229	0.939708	0.027541	0.124656	0.007937	0.328949	0.358266	1	0.156577	0.999252	0.69412	0.347244
**MDA-MB-468**	0.834236	0.157308	0.024946	0.1726	0.458817	0.196325	0.181082	0.156577	1	0.156744	0.160088	0.917797
**MCF7**	0.124419	0.929278	0.027563	0.066447	0.008587	0.329076	0.357365	0.999252	0.156744	1	0.644436	0.34743
**SK-OV-3**	0.126848	0.735374	0.027667	0.165933	0.008507	0.364105	0.414241	0.69412	0.160088	0.644436	1	0.351646
**OVCAR-3**	0.780448	0.348156	0.023656	0.36712	0.532027	0.394676	0.377482	0.347244	0.917797	0.34743	0.351646	1
Subtelomere 9p	**U-2 OS**	**Saos-2**	**H295R**	**G-292**	**SK-LU-1**	**SUSM-1**	**VA-13**	**A-431**	**MDA-MB-468**	**MCF7**	**SK-OV-3**	**OVCAR-3**
**U-2 OS**	1	0.243135	0.069564	0.156895	0.048432	0.707627	0.817987	0.996382	0.195594	0.767365	0.035125	0.039556
**Saos-2**	0.243135	1	0.025958	0.385001	0.310606	0.258343	0.237431	0.24261	0.359307	0.254967	0.407183	0.008137
**H295R**	0.069564	0.025958	1	0.067451	0.049011	0.06951	0.070677	0.068432	0.067398	0.069389	0.072233	0.042528
**G-292**	0.156895	0.385001	0.067451	1	0.060374	0.206893	0.147888	0.162769	0.839819	0.195633	0.837559	0.036776
**SK-LU-1**	0.048432	0.310606	0.049011	0.060374	1	0.049886	0.050088	0.046302	0.05732	0.049311	0.071273	0.017928
**SUSM-1**	0.707627	0.258343	0.06951	0.206893	0.049886	1	0.526133	0.746246	0.263678	0.940427	0.059134	0.03935
**VA-13**	0.817987	0.237431	0.070677	0.147888	0.050088	0.526133	1	0.852575	0.177574	0.586253	0.034216	0.040474
**A-431**	0.996382	0.24261	0.068432	0.162769	0.046302	0.746246	0.852575	1	0.205798	0.799098	0.05145	0.038703
**MDA-MB-468**	0.195594	0.359307	0.067398	0.839819	0.05732	0.263678	0.177574	0.205798	1	0.248681	0.652628	0.036911
**MCF7**	0.767365	0.254967	0.069389	0.195633	0.049311	0.940427	0.586253	0.799098	0.248681	1	0.055153	0.039294
**SK-OV-3**	0.035125	0.407183	0.072233	0.837559	0.071273	0.059134	0.034216	0.05145	0.652628	0.055153	1	0.040175
**OVCAR-3**	0.039556	0.008137	0.042528	0.036776	0.017928	0.03935	0.040474	0.038703	0.036911	0.039294	0.040175	1
Subtelomere 11q	**U-2 OS**	**Saos-2**	**G-292**	**SUSM-1**	**VA-13**	**A-431**	**MDA-MB-468**	**MCF7**	**SK-OV-3**	**OVCAR-3**		
**U-2 OS**	1	0.130209	0.105229	0.105744	0.139822	0.106336	0.146161	0.12393	0.124895	0.43649		
**Saos-2**	0.130209	1	0.441903	0.397517	0.49885	0.803934	0.938606	0.340615	0.353081	0.217053		
**G-292**	0.105229	0.441903	1	0.823374	0.728938	0.650995	0.145847	0.52125	0.580385	0.012434		
**SUSM-1**	0.105744	0.397517	0.823374	1	0.522111	0.592157	0.103165	0.674399	0.749806	0.011563		
**VA-13**	0.139822	0.49885	0.728938	0.522111	1	0.725776	0.155881	0.12762	0.16113	0.040256		

A-431	0.106336	0.803934	0.650995	0.592157	0.725776	1	0.81152	0.51049	0.528461	0.176473
MDA-MB-468	0.146161	0.938606	0.145847	0.103165	0.155881	0.81152	1	0.061373	0.06621	0.033442
MCF7	0.12393	0.340615	0.52125	0.674399	0.12762	0.51049	0.061373	1	0.867492	0.030254
SK-OV-3	0.124895	0.353081	0.580385	0.749806	0.16113	0.528461	0.06621	0.867492	1	0.030107
OVCAR-3	0.43649	0.217053	0.012434	0.011563	0.040256	0.176473	0.033442	0.030254	0.030107	1

Subtelomere 15q	U-2 OS	Saos-2	H295R	G-292	SK-LU-1	SUSM-1	VA-13	A-431	MDA-MB-468	MCF7	SK-OV-3	OVCAR-3
U-2 OS	1	0.528707	0.021477	0.077242	0.883403	0.46795	0.382914	0.286476	0.948856	0.859734	0.396838	0.267429
Saos-2	0.528707	1	0.020957	0.076024	0.452994	0.730178	0.651651	0.178542	0.586164	0.624504	0.653838	0.670765
H295R	0.021477	0.020957	1	0.020805	0.021583	0.016087	0.017792	0.021679	0.020925	0.021652	0.017128	0.02225
G-292	0.077242	0.076024	0.020805	1	0.112373	0.161712	0.117366	0.03996	0.104598	0.064789	0.129411	0.038022
SK-LU-1	0.883403	0.452994	0.021583	0.112373	1	0.428911	0.34568	0.346318	0.842695	0.743686	0.360625	0.220971
SUSM-1	0.46795	0.730178	0.016087	0.161712	0.428911	1	0.953396	0.267657	0.494019	0.518324	0.943739	0.941111
VA-13	0.382914	0.651651	0.017792	0.117366	0.34568	0.953396	1	0.202079	0.410088	0.43158	0.988439	0.875057
A-431	0.286476	0.178542	0.021679	0.03996	0.346318	0.267657	0.202079	1	0.322635	0.222495	0.217699	0.080139
MDA-MB-468	0.948856	0.586164	0.020925	0.104598	0.842695	0.494019	0.410088	0.322635	1	0.922192	0.422477	0.317597
MCF7	0.859734	0.624504	0.021652	0.064789	0.743686	0.518324	0.43158	0.222495	0.922192	1	0.444045	0.330423
SK-OV-3	0.396838	0.653838	0.017128	0.129411	0.360625	0.943739	0.988439	0.217699	0.422477	0.444045	1	0.867073
OVCAR-3	0.267429	0.670765	0.02225	0.038022	0.220971	0.941111	0.875057	0.080139	0.317597	0.330423	0.867073	1

Subtelomere 16q	U-2 OS	Saos-2	H295R	G-292	SK-LU-1	SUSM-1	VA-13	A-431	MDA-MB-468	MCF7	SK-OV-3	OVCAR-3
U-2 OS	1	0.361696	0.012213	0.514042	0.415736	0.462833	0.24613	0.434316	0.893185	0.460222	0.619382	0.063008
Saos-2	0.361696	1	0.39528	0.296451	0.46905	0.28758	0.244798	0.28018	0.380172	0.286882	0.429944	0.950053
H295R	0.012213	0.39528	1	0.021028	0.034758	0.018395	0.018351	0.014154	0.013488	0.017828	0.018404	0.128692
G-292	0.514042	0.296451	0.021028	1	0.008103	0.804878	0.091029	0.736151	0.398809	0.798798	0.177858	0.102586
SK-LU-1	0.415736	0.46905	0.034758	0.008103	1	0.016206	0.001381	0.044974	0.4688	0.019474	0.694241	0.169287
SUSM-1	0.462833	0.28758	0.018395	0.804878	0.016206	1	0.201813	0.885972	0.355845	0.987268	0.153722	0.088226
VA-13	0.24613	0.244798	0.018351	0.091029	0.001381	0.201813	1	0.391885	0.185249	0.224567	0.081022	0.092322
A-431	0.434316	0.28018	0.014154	0.736151	0.044974	0.885972	0.391885	1	0.334119	0.898096	0.145859	0.065391

**MDA-MB-468**	0.893185	0.380172	0.013488	0.398809	0.4688	0.355845	0.185249	0.334119	1	0.353753	0.707871	0.069143
**MCF7**	0.460222	0.286882	0.017828	0.798798	0.019474	0.987268	0.224567	0.898096	0.353753	1	0.152431	0.085073
**SK-OV-3**	0.619382	0.429944	0.018404	0.177858	0.694241	0.153722	0.081022	0.145859	0.707871	0.152431	1	0.093048
**OVCAR-3**	0.063008	0.950053	0.128692	0.102586	0.169287	0.088226	0.092322	0.065391	0.069143	0.085073	0.093048	1

ALT+ samples are marked in blue; ALT- samples are marked in red. The values in the cells represent the P value of the two compared samples. Significant values ($P < 0.05$) are marked in yellow.

Table S6. P values of a two-tailed Mann–Whitney U-test comparing H3K9me3 levels in ALT+ samples and FSE WT cells.

**Subtelomere 2p Promoter**	**U-2 OS**	**Saos-2**	**G-292**	**SK-LU-1**	**SUSM-1**	**FSE**
**U-2 OS**	1	0.665006	0.029401	0.030383	0.112351	0.030383
**Saos-2**	0.665006	1	0.029401	0.030383	0.193931	0.030383
**G-292**	0.029401	0.029401	1	0.029401	0.029401	0.029401
**SK-LU-1**	0.030383	0.030383	0.029401	1	0.060602	0.665006
**SUSM-1**	0.112351	0.193931	0.029401	0.060602	1	0.030383
FSE	0.030383	0.030383	0.029401	0.665006	0.030383	1
**Subtelomere 5p Promoter**	**U-2 OS**	**Saos-2**	**G-292**	**SK-LU-1**	**SUSM-1**	**FSE**
**U-2 OS**	1	0.030383	0.030383	0.030383	0.030383	0.030383
**Saos-2**	0.030383	1	0.885234	0.030383	0.030383	0.030383
**G-292**	0.030383	0.885234	1	0.030383	0.030383	0.030383
**SK-LU-1**	0.030383	0.030383	0.030383	1	0.060602	0.193931
**SUSM-1**	0.030383	0.030383	0.030383	0.060602	1	0.030383
FSE	0.030383	0.030383	0.030383	0.193931	0.030383	1
**Subtelomere 10q\13q\19q Promoters**	**U-2 OS**	**Saos-2**	**G-292**	**SK-LU-1**	**SUSM-1**	**FSE**
**U-2 OS**	1	0.030383	0.193931	0.030383	0.030383	0.030383
**Saos-2**	0.030383	1	0.885234	0.030383	0.030383	0.030383
**G-292**	0.193931	0.885234	1	0.030383	0.060602	0.030383
**SK-LU-1**	0.030383	0.030383	0.030383	1	0.112351	0.665006
**SUSM-1**	0.030383	0.030383	0.060602	0.112351	1	0.470486
FSE	0.030383	0.030383	0.030383	0.665006	0.470486	1
**Subtelomere 2p Distal Region**	**U-2 OS**	**Saos-2**	**G-292**	**SK-LU-1**	**SUSM-1**	**FSE**
**U-2 OS**	1	0.030383	0.193931	0.312321	0.060602	0.105193
**Saos-2**	0.030383	1	0.885234	0.030383	0.030383	0.105193
**G-292**	0.193931	0.885234	1	0.112351	0.030383	0.105193
**SK-LU-1**	0.312321	0.030383	0.112351	1	0.885234	0.24716
**SUSM-1**	0.060602	0.030383	0.030383	0.885234	1	0.105193
FSE	0.105193	0.105193	0.105193	0.24716	0.105193	1
**Subtelomere 2q/10q/13q Distal Regions**	**U-2 OS**	**Saos-2**	**G-292**	**SK-LU-1**	**SUSM-1**	**FSE**
**U-2 OS**	1	0.030383	0.112351	0.112351	0.030383	0.105193
**Saos-2**	0.030383	1	0.470486	0.312321	0.030383	0.105193
**G-292**	0.112351	0.470486	1	0.193931	0.060602	0.105193
**SK-LU-1**	0.112351	0.312321	0.193931	1	0.312321	0.24716
**SUSM-1**	0.030383	0.030383	0.060602	0.312321	1	0.105193
FSE	0.105193	0.105193	0.105193	0.24716	0.105193	1
**Subtelomere 5p Distal Region**	**U-2 OS**	**Saos-2**	**G-292**	**SK-LU-1**	**SUSM-1**	**FSE**
**U-2 OS**	1	0.112351	0.030383	0.060602	0.030383	0.105193
**Saos-2**	0.112351	1	0.060602	0.193931	0.030383	0.105193
**G-292**	0.030383	0.060602	1	0.470486	0.885234	0.105193
**SK-LU-1**	0.060602	0.193931	0.470486	1	0.312321	0.105193

SUSM-1	0.030383	0.030383	0.885234	0.312321	1	0.105193
FSE	0.105193	0.105193	0.105193	0.105193	0.105193	1
**Subtelomere 7q Distal Region**	**U-2 OS**	**Saos-2**	**G-292**	**SK-LU-1**	**SUSM-1**	**FSE**
U-2 OS	1	0.030383	0.030383	0.030383	0.030383	0.105193
Saos-2	0.030383	1	0.665006	0.112351	0.470486	0.105193
G-292	0.030383	0.665006	1	0.112351	0.665006	0.105193
SK-LU-1	0.030383	0.112351	0.112351	1	0.193931	0.24716
SUSM-1	0.030383	0.470486	0.665006	0.193931	1	0.105193
FSE	0.105193	0.105193	0.105193	0.24716	0.105193	1
**Subtelomere 15q Distal Region**	**U-2 OS**	**Saos-2**	**G-292**	**SK-LU-1**	**SUSM-1**	**FSE**
U-2 OS	1	0.312321	0.885234	0.05183	0.030383	0.105193
Saos-2	0.312321	1	0.665006	0.05183	0.030383	0.105193
G-292	0.885234	0.665006	1	0.05183	0.060602	0.105193
SK-LU-1	0.05183	0.05183	0.05183	1	0.05183	0.77283
SUSM-1	0.030383	0.030383	0.060602	0.05183	1	0.105193
FSE	0.105193	0.105193	0.105193	0.77283	0.105193	1

ALT+ samples are marked in blue; FSE sample is marked in gray. The values in the cells represent the P value of the two compared samples. Significant values ($P < 0.05$) are marked in yellow.

Table S7. P values of a two-tailed Mann–Whitney U-test comparing H3K4me3 levels in ALT+ samples and FSE WT cells.

**Subtelomere 2p Promoter**	**U-2 OS**	**Saos-2**	**G-292**	**SK-LU-1**	**SUSM-1**	**FSE**
U-2 OS	1	0.030383	0.029401	0.060602	0.030383	0.470486
Saos-2	0.030383	1	0.309424	0.030383	0.030383	0.112351
G-292	0.029401	0.309424	1	0.029401	0.029401	0.059072
SK-LU-1	0.060602	0.030383	0.029401	1	0.112351	0.112351
SUSM-1	0.030383	0.030383	0.029401	0.112351	1	0.030383
FSE	0.470486	0.112351	0.059072	0.112351	0.030383	1
**Subtelomere 5p Promoter**	**U-2 OS**	**Saos-2**	**G-292**	**SK-LU-1**	**SUSM-1**	**FSE**
U-2 OS	1	0.030383	0.030383	0.060602	0.112351	0.060602
Saos-2	0.030383	1	0.112351	0.030383	0.030383	0.030383
G-292	0.030383	0.112351	1	0.030383	0.030383	0.030383
SK-LU-1	0.060602	0.030383	0.030383	1	0.885234	0.470486
SUSM-1	0.112351	0.030383	0.030383	0.885234	1	0.665006
FSE	0.060602	0.030383	0.030383	0.470486	0.665006	1
**Subtelomere 10q\13q\19q Promoters**	**U-2 OS**	**Saos-2**	**G-292**	**SK-LU-1**	**SUSM-1**	**FSE**
U-2 OS	1	0.030383	0.030383	0.030383	0.665006	0.030383
Saos-2	0.030383	1	0.885234	0.030383	0.030383	0.112351
G-292	0.030383	0.885234	1	0.030383	0.030383	0.060602
SK-LU-1	0.030383	0.030383	0.030383	1	0.030383	0.030383
SUSM-1	0.665006	0.030383	0.030383	0.030383	1	0.030383
FSE	0.030383	0.112351	0.060602	0.030383	0.030383	1

Subtelomere 2p Distal Region	U-2 OS	Saos-2	G-292	SK-LU-1	SUSM-1	FSE
U-2 OS	1	0.030383	0.885234	0.885234	0.193931	0.665006
Saos-2	0.030383	1	0.060602	0.112351	0.030383	0.060602
G-292	0.885234	0.060602	1	0.665006	0.885234	0.470486
SK-LU-1	0.885234	0.112351	0.665006	1	0.665006	0.885234
SUSM-1	0.193931	0.030383	0.885234	0.665006	1	0.470486
FSE	0.665006	0.060602	0.470486	0.885234	0.470486	1
**Subtelomere 2q/10q/13q Distal Regions**	**U-2 OS**	**Saos-2**	**G-292**	**SK-LU-1**	**SUSM-1**	**FSE**
U-2 OS	1	0.030383	0.030383	0.665006	0.112351	0.885234
Saos-2	0.030383	1	0.312321	0.030383	0.030383	0.030383
G-292	0.030383	0.312321	1	0.030383	0.030383	0.030383
SK-LU-1	0.665006	0.030383	0.030383	1	0.193931	0.665006
SUSM-1	0.112351	0.030383	0.030383	0.193931	1	0.665006
FSE	0.885234	0.030383	0.030383	0.665006	0.665006	1
**Subtelomere 5p Distal Region**	**U-2 OS**	**Saos-2**	**G-292**	**SK-LU-1**	**SUSM-1**	**FSE**
U-2 OS	1	0.030383	0.030383	0.665006	0.193931	0.060602
Saos-2	0.030383	1	0.193931	0.030383	0.030383	0.030383
G-292	0.030383	0.193931	1	0.030383	0.030383	0.030383
SK-LU-1	0.665006	0.030383	0.030383	1	0.885234	0.060602
SUSM-1	0.193931	0.030383	0.030383	0.885234	1	0.112351
FSE	0.060602	0.030383	0.030383	0.060602	0.112351	1
**Subtelomere 7q Distal Region**	**U-2 OS**	**Saos-2**	**G-292**	**SK-LU-1**	**SUSM-1**	**FSE**
U-2 OS	1	0.030383	0.030383	0.665006	0.312321	0.885234
Saos-2	0.030383	1	0.112351	0.030383	0.030383	0.030383
G-292	0.030383	0.112351	1	0.030383	0.030383	0.030383
SK-LU-1	0.665006	0.030383	0.030383	1	0.060602	0.665006
SUSM-1	0.312321	0.030383	0.030383	0.060602	1	0.112351
FSE	0.885234	0.030383	0.030383	0.665006	0.112351	1
**Subtelomere 15q Distal Region**	**U-2 OS**	**Saos-2**	**G-292**	**SK-LU-1**	**SUSM-1**	**FSE**
U-2 OS	1	0.193931	0.030383	0.312321	0.193931	0.312321
Saos-2	0.193931	1	0.030383	0.312321	0.885234	0.665006
G-292	0.030383	0.030383	1	0.030383	0.030383	0.030383
SK-LU-1	0.312321	0.312321	0.030383	1	0.312321	0.885234
SUSM-1	0.193931	0.885234	0.030383	0.312321	1	0.665006
FSE	0.312321	0.665006	0.030383	0.885234	0.665006	1

ALT+ samples are marked in blue; FSE sample is marked in gray. The values in the cells represent. the P value of the two compared samples. Significant values ($P < 0.05$) are marked in yellow.
